# Cyclic connectivity index of fuzzy incidence graphs with applications in the highway system of different cities to minimize road accidents and in a network of different computers

**DOI:** 10.1371/journal.pone.0257642

**Published:** 2021-09-21

**Authors:** Irfan Nazeer, Tabasam Rashid, Muhammad Tanveer Hussain

**Affiliations:** Department of Mathematics, University of Management and Technology, Lahore, Pakistan; Al Mansour University College-Baghdad-Iraq, IRAQ

## Abstract

A parameter is a numerical factor whose values help us to identify a system. Connectivity parameters are essential in the analysis of connectivity of various kinds of networks. In graphs, the strength of a cycle is always one. But, in a fuzzy incidence graph (FIG), the strengths of cycles may vary even for a given pair of vertices. Cyclic reachability is an attribute that decides the overall connectedness of any network. In graph the cycle connectivity (*CC*) from vertex *a* to vertex *b* and from vertex *b* to vertex *a* is always one. In fuzzy graph (FG) the CC from vertex *a* to vertex *b* and from vertex *b* to vertex *a* is always same. But if someone is interested in finding *CC* from vertex *a* to an edge *ab*, then graphs and FGs cannot answer this question. Therefore, in this research article, we proposed the idea of *CC* for FIG. Because in FIG, we can find *CC* from vertex *a* to vertex *b* and also from vertex *a* to an edge *ab*. Also, we proposed the idea of *CC* of fuzzy incidence cycles (FICs) and complete fuzzy incidence graphs (*CFIGs*). The fuzzy incidence cyclic cut-vertex, fuzzy incidence cyclic bridge, and fuzzy incidence cyclic cut pair are established. A condition for *CFIG* to have fuzzy incidence cyclic cut-vertex is examined. Cyclic connectivity index and average cyclic connectivity index of FIG are also investigated. Three different types of vertices, such as cyclic connectivity increasing vertex, cyclically neutral vertex and, cyclic connectivity decreasing vertex, are also defined. The real-life applications of *CC* of FIG in a highway system of different cities to minimize road accidents and a computer network to find the best computers among all other computers are also provided.

## 1 Introduction

Graphs are convenient tools to explain associations between different types of entities under examination. Vertices or nodes denote entities, and edges or arcs explain the vertices’ connections. A mathematical structure to describe unpredictability and equivocacy in daily life circumstances was first addressed by Zadeh. He presented the perception of the fuzzy set (FS) [1]. He aimed to develop a mathematical theory to tackle unreliability and inexactness. The major difference between sets and FSs is that the sets classified the universal set (US) into two values: members and non-members. On the other hand, FS designates a sequence of membership values to elements of the US from [0, 1] closed interval. Also, FSs are beneficial to learn about quality variables, including reasoning, prettiness, uniformity, etc. There is a deficiency in describing the entities and their association, and we need to draw a FG model. Rosenfeld [2] was the first who proposed the crucial idea of FGs after the revolutionary work of Zadeh’s on FSs.

FGs can be expressed as the strength of relationships between objects. The study of FGs plays an essential role for many students as well as mathematicians to take part in this field, such as Bhutani and Rosenfeld [3] suggested the notion of geodesics in FGs. An arc of a fuzzy graph is called strong if its weight is at least as great as the strength of connectedness of its end nodes when it is deleted; this idea was provided by Bhutani and Rosenfeld [4]. The notion of fuzzy end nodes in FGs was presented by Bhutani and Rosenfeld [5]. Gani and Ahamad [6] presented a way to compute different types of degrees, order, and size of FGs and compare the relationship among degree, order, and size of FGs. Bhutani et al. [7] explained degrees of end nodes and cut-nodes in FGs. Al-Hawary [8] described different kinds of operations on FGs. Moreover, he introduced and studied the notion of balanced FG and gave necessary and sufficient conditions for the preceding products of two fuzzy balanced graphs to be balanced. Al-Hawary and Horani [9] provided three new products on product FGs and gave sufficient conditions for each one of them to be strong. They also discussed balanced and co-balanced product FGs. Al-Hawary [10] investigated several classes of FGs and provided two new operations on FGs, such as parallel connection and series connection. He also showed that parallel connection and series connection of balanced FGs need not be balanced. Woznaik et al. [11] presented electronic modules, infrastructure, and fuzzy rules control model with implemented software for new generation home environment. Zielonka et al. [12] presented IoT convection installation for a small house with the developed remote platform control system. Connectivity analysis of cyclically balanced FGs was a fascinating idea provided by Jicy, and Mathew [13]. Binu et al. [14, 15] gave the idea to calculate the connectivity index and Wiener index of a FG and its applications to human trafficking and illegal immigration networks, respectively. The concept of *CC* of a FG, *CC* of fuzzy trees, fuzzy cycles, and complete FGs was examined by Mathew and Sunitha [16]. They also initiated the idea of cyclic cut nodes and cyclic bridges in FGs. Mathew and Sunitha [17] provided the concept of node-strength sequence, fuzzy node connectivity, and fuzzy arc connectivity. They also furnished a new clustering technique based on fuzzy arc connectivity. The criterion for connectivity of a FG and the structure of the complement of a fuzzy cycle was investigated by Narayan and Sunitha [18]. Bipolar FGs, various methods of their construction, the idea of isomorphism of bipolar FGs, and some of their key properties were proposed by Akram [19]. Akram and Alshehri [20] proposed an idea of intuitionistic fuzzy cycles and intuitionistic fuzzy trees. Depending on the strength of an arc, Mathew and Sunitha [21] classified arcs of a FG into three different types, namely *α*-strong, *β*-strong, and *δ*-arc. Mathew and Sunitha [22] initiated the idea of the strongest strong cycle and *θ*-FGs. Different kinds of operations, including Cartesian product, composition, union, and join in FGs, were given by Mordeson and Chang-Shyh [23]. Fuzzy tolerance, fuzzy tolerance graph, fuzzy bounded tolerance graph, fuzzy interval containment graph and regular representation of fuzzy tolerance graph, fuzzy unit tolerance graph, and proper tolerance graph were discussed by Samanta and Pal [24]. A fuzzy planar graph is a very important subclass of FG that was presented by Samanta and Pal [25]. Mordeson and Nair [26] brought the idea of fuzzy hypergraphs. Three kinds of operations, including direct product, semi-strong product, and strong product for interval-valued FGs was provided by Rashmanlou, and Jun [27]. Sunitha and Vijayakumar [28] defined complement of a FG. Mordeson and Nair [29] introduced and examined the concepts of chords, twigs, 1-chains with boundary zero, cycle vectors, coboundary, and cocycles for FGs. They have also shown that although the set of cycle vectors, fuzzy cycle vectors, cocycles, and fuzzy cocycles do not necessarily form vector spaces over the field *Z*_2_ of integers modulo 2, they nearly do. Later on, different mathematicians participated in the development of graphs and FGs. Their achievements can be seen in [30–35].

There is a flaw in FGs because they do not give any clue of the impact of a vertex on edge. This lack of FGs become the fundamental cause to establish the scheme of FIG. The proposal of FIGs was first initiated by Dinesh [36]. For example, in a highway system, if vertices represent various cites and edges serve as highways, introducing the degree of connection between city *L* and the highway *LM* joining cities *L* and *M* permits a profound analysis of the highway system. This connection could be the ramp system joining *L* and *LM*. We indicate this relationship by the ordered pair (*L*, *LM*). Malik et al. [37] applied FIGs in different types of applications. Mathew and Mordeson [38] proposed the idea of cut pairs and fuzzy incidence trees in FIGs. They also discussed some vital properties of FIGs. Three different types of nodes, including fuzzy incidence connectivity enhancing node, fuzzy incidence connectivity reducing node, and fuzzy incidence connectivity neutral node in FIGs was introduced by Fang et al. [39]. Like node and edge connectivity in graphs, Mathew et al. [40] discussed these concepts for FIGs. Mordeson and Mathew [41] developed fuzzy end nodes and fuzzy incidence cut vertices in FIGs. Nazeer et al. [42] presented the idea of intuitionistic fuzzy incidence graphs (IFIGs) as a generalization of FIGs along with their certain properties. They introduced a variety of operations in IFIGs. They also provided a fascinating application of the product of IFIGs. The idea of order, size, domination, strong fuzzy incidence domination, and weak fuzzy incidence domination in FIGs was proposed by Nazeer et al. [43]. Nazeer and Rashid [44] presented the idea of picture FIGs. They introduced picture fuzzy cut-vertices, picture fuzzy bridges, picture fuzzy incidence cut pairs, and picture fuzzy incidence cut-vertices. More extensive and comprehensive work on FIGs, can be seen [45–48].

Connectivity parameters are connectivity measures of any system. In graphs, the connectivity between any two vertices is 1, and in FGs, it is from closed interval [0, 1]. There are certain motives to propose the concept of *CC* in FIGs. Firstly, in FGs, we can only compute the *CC* from vertex *l* to vertex *m* and from vertex *m* to the vertex *l* but if someone is interested in examining the *CC* from vertex *l* to an edge *lm*, then FGs are not enough to answer this question. Therefore, we propose the concept of *CC* in FIGs because FIGs permit us to find the *CC* from vertex *l* to an edge *lm* due to the presence of an incidence pair in FIGs. Secondly, in FIGs, the *CC* from vertex *l* to an edge *lm* and vertex *m* to an edge *lm* may or may not be the same. Thirdly, we cannot apply graphs and FGs to the applications of the highway systems of different cities and networks of different computers provided in Section 5 due to the non-availability of the influence of a vertex on and edge. Fourthly, the objective to introduce these ideas to FIGs is that Mathew and Sunitha [16] initiated the notion of *CC* in FGs. Later, Binu et al. [49] initiated an idea of cyclic connectivity index (*CCI*) and average cyclic connectivity index (*ACCI*) of FGs. We extended their work for FIGs. This paper establishes *CC*, *CCI* and *ACCI* of FIGs.

The other part of this article is constructed as follows. Section 2 consists of some introductory outcomes essential to comprehend the remaining portion of the article. *CC*, fuzzy incidence cyclic cut-vertex (*FICCV*), fuzzy incidence cyclic bridge (*FICB*) and fuzzy incidence cyclic cut pair (*FICCP*) of FIG are explained in Section 3. The formula to determine *CCI*, the way to manipulate *ACCI* of FIG, and three different types of vertices, namely, cyclic connectivity increasing vertex (*CCIV*), cyclically neutral vertex (*CNV*), and cyclic connectivity decreasing vertex (*CCDV*) are described in Section 4. The real-life applications of *CC* of a FIG in a highway system of different cities to reduce road accidents and a computer network to find the best computers sharing the maximum amount of data among all other computers are discussed in Section 5. A comparative analysis of our study with the existing study is provided in Section 6. Section 7 carries some conclusions and future directions.

## 2 Preliminaries

This section carries some elementary and rudimentary definitions and results of FIGs. These will be useful to understand the contents of the article. ∧ indicates the minimum operator, and ∨ denotes the maximum operator in this article.

**Definition 1**. [41] *A fuzzy subset (FSS) of a set is a function of the set into the closed interval* [0, 1]. *A FG on* (*V*, *E*) *is a pair* (*σ*, *τ*), *where σ is a FSS of V and τ is a FSS of E such that for every l*, *m* ∈ *V*, *τ*(*lm*) ≤ *σ*(*l*) ∧ *σ*(*m*).

**Definition 2**. [41, *Definition 2.1*] *Let G* = (*V*, *E*, *I*), *where I* ⊆ *V* × *E*. *Then G is called an incidence graph (IG)*.

**Definition 3**. [41] *Let G* = (*V*, *E*, *I*) *be an IG. A sequence of distinct vertices P*_1_: *k*_0_, (*k*_0_, *k*_0_ *k*_1_), *k*_0_ *k*_1_, (*k*_1_, *k*_0_ *k*_1_), *k*_1_, …, *k*_*n*−1_, (*k*_*n*−1_, *k*_*n*−1_ *k*_*n*_), *k*_*n*−1_ *k*_*n*_, (*k*_*n*_, *k*_*n*−1_ *k*_*n*_), *k*_*n*_ *is called an incidence path and vertices* *k*_0_ *and* *k*_*n*_ *are said to be connected. The incidence strength* (*I*_*s*_) *of* *P*_1_ *is defined as η*(*k*_0_, *k*_0_ *k*_1_) ∧ *η*(*k*_1_, *k*_0_ *k*_1_) ∧ … ∧ *η*(*k*_*n*−1_, *k*_*n*−1_ *k*_*n*_) *and is expressed by* $\left(I_{s_{P_{1}}}\right)$. *A sequence P*_2_: *k*_0_, (*k*_0_, *k*_0_ *k*_1_), *k*_0_ *k*_1_, (*k*_1_, *k*_0_ *k*_1_), *k*_1_, …, *k*_*n*−1_, (*k*_*n*−1_, *k*_*n*−1_ *k*_*n*_), *k*_*n*−1_ *k*_*n*_, (*k*_*n*_, *k*_*n*−1_ *k*_*n*_), *k*_*n*_, (*k*_*n*_, *k*_*n*_ *k*_*n*+1_), *k*_*n*_ *k*_*n*+1_ *is another incidence path between k*_0_ *and k*_*n*_ *k*_*n*+1_. *The I*_*s*_ *of P*_2_ *is defined as η*(*k*_0_, *k*_0_ *k*_1_) ∧ *η*(*k*_1_, *k*_0_ *k*_1_) ∧ … ∧ *η*(*k*_*n*_, *k*_*n*_ *k*_*n*+1_). *Let l*, *m* ∈ *V* ∪ *E*. *We define* $ICONN_{\tilde{G}}\left(l,m\right)$ *to be the I*_*s*_ *of the strongest path from l to m*.

**Definition 4**. [41, *Definition 2.9*] *Let G* = (*σ*, *τ*), *where σ is a FSS of V and τ is a FSS of E*. *If η is a fuzzy incidence of G, then* $\tilde{G}=\left(\sigma ,\tau ,\eta \right)$ *is said to be a FIG*.

**Fig 1:**
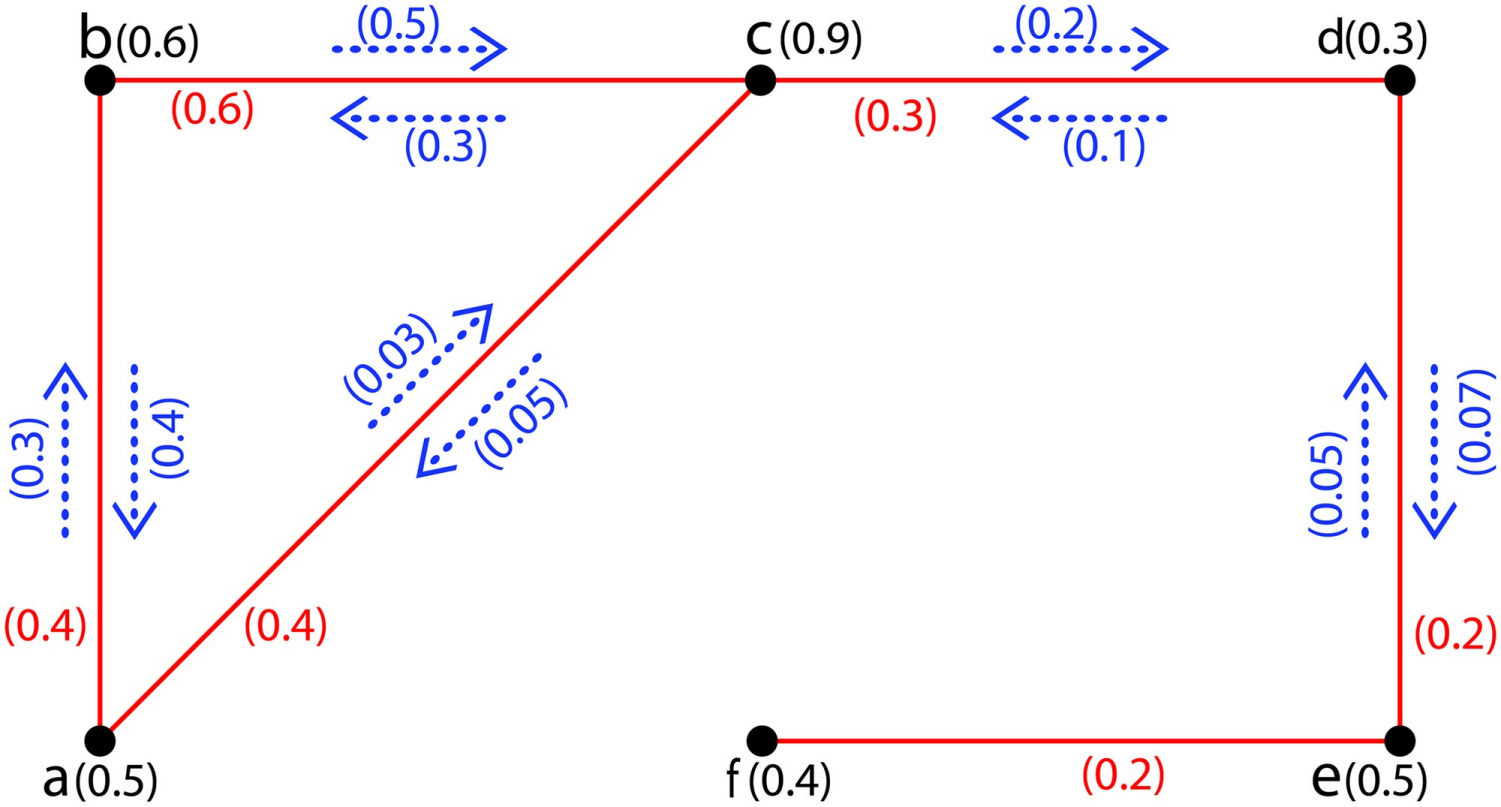
A FIG.

**Example 1**. *In*Fig 1*a FIG*$\tilde{G}=\left\{\sigma ,\tau ,\eta \right\}$is shown with σ* = {*a*, *b*, *c*, *d*, *e*, *f*} *and σ*(*a*) = 0.5, *σ*(*b*) = 0.6, *σ*(*c*) = 0.9, *σ*(*d*) = 0.3, *σ*(*e*) = 0.5, *σ*(*f*) = 0.4*τ*(*ab*) = 0.4, *τ*(*ac*) = 0.4, *τ*(*bc*) = 0.6, *τ*(*cd*) = 0.3, *τ*(*de*) = 0.2, *τ*(*ef*) = 0.2; *η*(*a*, *ab*) = 0.3, *η*(*b*, *ab*) = 0.4, *η*(*a*, *ac*) = 0.03, *η*(*c*, *ac*) = 0.05, *η*(*b*, *bc*) = 0.5, *η*(*c*, *bc*) = 0.3, *η*(*c*, *cd*) = 0.2, *η*(*d*, *cd*) = 0.1, *η*(*d*, *de*) = 0.07, *η*(*e*, *de*) = 0.05, *η*(*e*, *ef*) = 0, *η*(*f*, *ef*) = 0. *There are two possible incidence paths from vertex a to d namely*, *P*_1_: *a*, (*a*, *ab*), *ab*, (*b*, *ab*), *b*, (*b*, *bc*), *bc*, (*c*, *bc*), *c*, (*c*, *cd*), *cd*, (*d*, *cd*), *dandP*_2_: *a*, (*a*, *ac*), *ac*, (*c*, *ac*), *c*, (*c*, *cd*), *cd*, (*d*, *cd*), *d with*$I_{s_{P_{1}}}=\eta \left(a,ab\right)\wedge \eta \left(b,ab\right)\wedge \eta \left(b,bc\right)\wedge \eta \left(c,bc\right)\wedge \eta \left(c,cd\right)\wedge \eta \left(d,cd\right)=0.3\wedge 0.4\wedge 0.3\wedge 0.5\wedge 0.3\wedge 0.2\wedge 0.1=0.1$. *Similarly*, $I_{s_{P_{2}}}=0.03$*and the strength of the strongest path from a to d is*$ICONN_{\tilde{G}}\left(a,d\right)=\vee \left\{I_{s_{P_{1}}},I_{s_{P_{2}}}=0.1,0.03=0.1\right\}$.

**Definition 5**. [38] *Any l* ∈ *V is said to be in the support of σ* (*Supp*(*σ*)) *if σ*(*l*) > 0, *lm* ∈ *E is said to be in the support of τ* (*Supp*(*τ*)) *if τ*(*lm*) > 0 *and* (*l*, *lm*) ∈ *V* × *E is said to be in the support of η* (*Supp*(*η*)) *if* *η*(*l*, *lm*) > 0. The σ*, τ* and η* *are indicating the support of σ*, *τ and η respectively. Any edge lm is an edge of the FIG* $\tilde{G}=\left(\sigma ,\tau ,\eta \right)$ *if* *lm* ∈ τ*. *Similarly, any* (*l*, *lm*) *is a incidence pair* (*I*_*p*_) *of* $\tilde{G}$ *if* (*l*, *lm*) ∈ η*.

**Definition 6**. [41, *Definition 2.11*] *Let*$\tilde{G}=\left(\sigma ,\tau ,\eta \right)$*be a FIG. Then an IG*$\tilde{H}=\left(\iota ,\kappa ,\varsigma \right)$*is known as partial fuzzy incidence subgraph* (*PFISG*) *of* $\tilde{G}$ *if ι* ⊆ *σ*, *κ* ⊆ *τ and* ς⊆η. $\tilde{H}$ *is named as fuzzy incidence subgraph* (*FISG*) *of* $\tilde{G}$ *if ι* = *σ*, *κ* = *τ and* ς=η.

**Definition 7**. [38, *Definition 3*] *If* (σ*, τ*, η*) *is a cycle then the FIG* $\tilde{G}=\left(\sigma ,\tau ,\eta \right)$ *is a cycle. If* (σ*, τ*, η*) *is a cycle and* ∄ *a single lm* ∈ τ* *such that τ*(*lm*) = ∧{*τ*(*uv*)|*uv* ∈ τ*} *then the FIG* $\tilde{G}$ *is a fuzzy cycle. The FIG* $\tilde{G}=\left(\sigma ,\tau ,\eta \right)$ *is a fuzzy incidence cycle (FIC), and* ∄ *a single* (*l*, *lm*) ∈ η* *such that* *η*(*l*, *lm*) = ∧{*η*(*u*, *uv*)|(*u*, *uv*) ∈ η*}.

**Definition 8**. [38, *Definition 7*] *Consider a FIG*$\tilde{G}=\left(\sigma ,\tau ,\eta \right)$. *Then an I*_*p*_ (*l*, *lm*) *is said to be a strong incidence pair* $\left(I_{p}^{s}\right)$ *if* $\eta \left(l,lm\right)\geq ICONN_{\tilde{G}-\left(l,lm\right)}$ *where* $ICONN_{\tilde{G}-\left(l,lm\right)}$ *indicates the maximum incidence strength of l* − *lm*. *Particularly, it is said to be* $\alpha -I_{p}^{s}$ *if* $\eta \left(l,lm\right)>ICONN_{\tilde{G}-\left(l,lm\right)}$ *and* $\beta -I_{p}^{s}$ *if* $\eta \left(l,lm\right)=ICONN_{\tilde{G}-\left(l,lm\right)}$. *A pair is said to be* $I_{p}^{s}$ *if it is either* $\alpha -I_{p}^{s}$ *or* $\beta -I_{p}^{s}$.

**Definition 9**. [38, *Definition 8*] *Consider a FIG*$\tilde{G}=\left(\sigma ,\tau ,\eta \right)$. *If*$\eta \left(l,lm\right)<ICONN_{\tilde{G}-\left(l,lm\right)}$*then* (*l*, *lm*) *is called δ*-*incidence pair* (*δ* − *IPr*).

**Proposition 1**. [38, *Proposition 1*] *Each FIC is a strong cycle*.

**Definition 10**. [38] *If η*(*l*, *lm*) = *σ*(*l*) ∧ *τ*(*lm*) *for each* (*l*, *lm*) ∈ η* *then a FIG* $\tilde{G}=\left(\sigma ,\tau ,\eta \right)$ *is named as a CFIG*.

**Proposition 2**. [38, *Proposition 9*] *A CFIG has no δ* − *IPr*.

## 3 Cycle connectivity of fuzzy incidence graphs

In this section, we present the novel idea of connectivity named as *CC* of a FIG. The *CC* of any FIG is denoted by Ω. The idea of Ω of a FIC is also illuminated. It is also proved that the Ω of a FIC $\tilde{G}$ is equal to the *I*_*s*_ of $\tilde{G}$. We have also introduced three innovative ideas, namely *FICCV*, *FICB*, and *FICCP*. On deleting the *FICCV*, *FICB*, and *FICCP* from the FIG, there will be an effect in the connectivity and Ω of a FIG. For easiness, in the coming sections, we will take *σ*(*a*) = 1 for every *a* ∈ σ* unless otherwise specifies.

**Definition 11**. *Assume*$\tilde{G}=\left\{\sigma ,\tau ,\eta \right\}$*is a FIC. The I*_*s*_*of FIC is the lowest η value of all I*_*p*_*in it*.

**Definition 12**. *Let*$\tilde{G}=\left\{\sigma ,\tau ,\eta \right\}$*be a FIG. Then for any two vertices l and m of*$\tilde{G}$, *there associated a set say ν*(*l*, *m*) *named the ν* − *estimation of l and m and is defined as ν*(*l*, *m*) = {*χ*: *χ* ∈ (0, 1]} *where χ is the I*_*s*_ *of a FIC passing through l and m*.

**Definition 13**. *Consider a FIG*$\tilde{G}=\left\{\sigma ,\tau ,\eta \right\}$. *Then* Ω *between l and m is defined as* ∨{*χ*: *χ* ∈ *ν*(*l*, *m*)|*l*, *m* ∈ σ*} *and it is indicated by* $\Omega_{l,m}^{\tilde{G}}$.

**Remark 1**. *If ν*(*l*, *m*) = ∅ *for some pair of vertices l and m, then* $\Omega_{l,m}^{\tilde{G}}\left(l,m\right)=0$.

**Fig 2:**
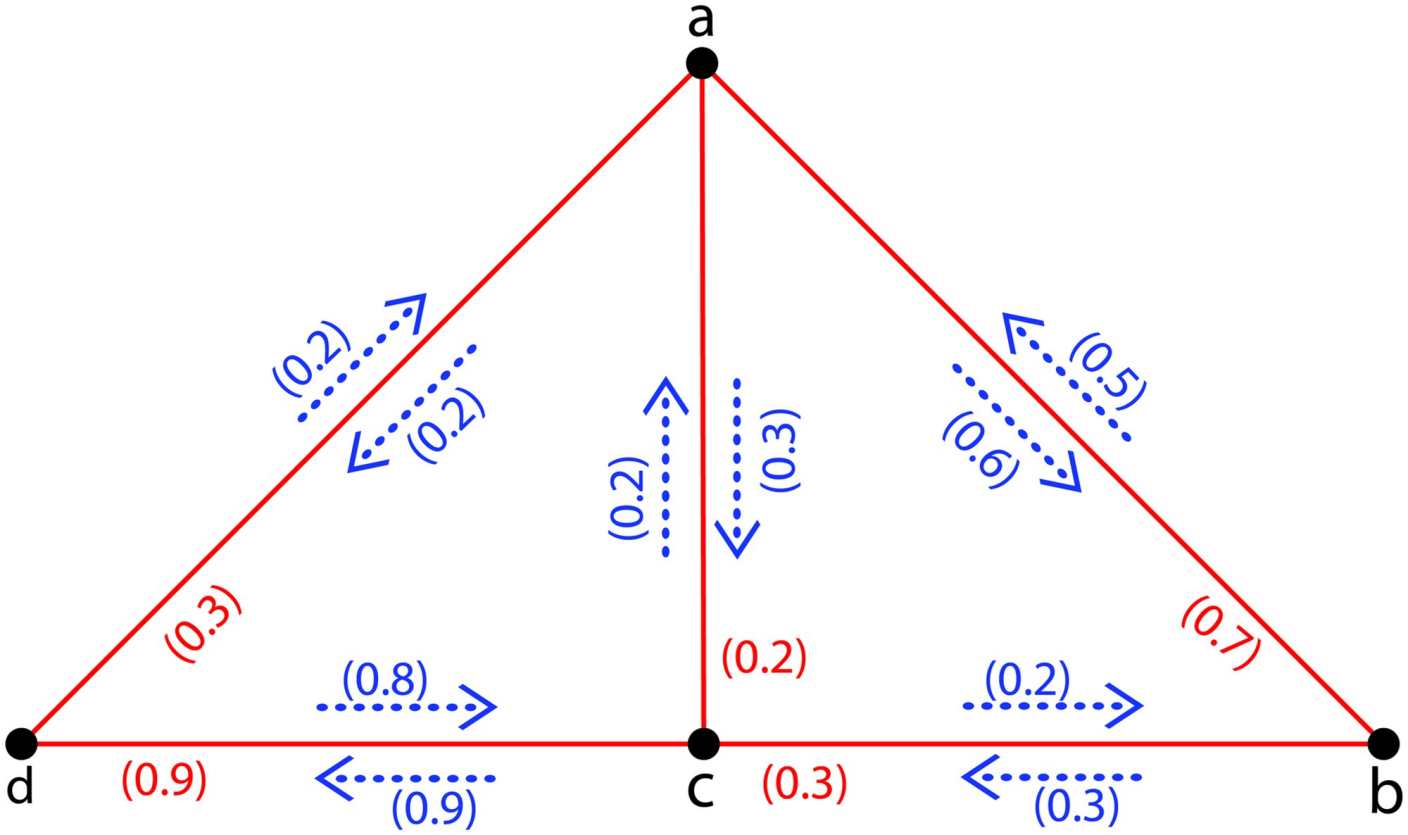
A FIG with $\Omega_{a,c}^{\tilde{G}}=0.2$.

**Example 2**. *In*Fig 2*a FIG*$\tilde{G}=\left\{\sigma ,\tau ,\eta \right\}$is shown with σ* = {*a*, *b*, *c*, *d*} *and τ*(*ab*) = 0.7, *τ*(*ac*) = 0.3, *τ*(*ad*) = 0.3, *τ*(*bc*) = 0.3, *τ*(*cd*) = 0.9; *η*(*a*, *ab*) = 0.6, *η*(*b*, *ab*) = 0.5, *η*(*a*, *ac*) = 0.2, *η*(*c*, *ac*) = 0.2, *η*(*a*, *ad*) = 0.2, *η*(*d*, *ad*) = 0.2, *η*(*b*, *bc*) = 0.3, *η*(*c*, *bc*) = 0.2, *η*(*c*, *cd*) = 0.9, *η*(*d*, *cd*) = 0.8 *a FIG. Here in*$\tilde{G}$, *abca*, *abcda and adca are all FICs. There are three FICs passing through a and c comprising*, *abca*, *adca and abcda with I*_*s*_ = ∧{*η*(*a*, *ab*) = 0.6, *η*(*b*, *ab*) = 0.5, *η*(*b*, *bc*) = 0.3, *η*(*c*, *bc*) = 0.2, *η*(*c*, *ac*) = 0.2, *η*(*a*, *ac*) = 0.2} = 0.2; = ∧{*η*(*a*, *ad*) = 0.2, *η*(*d*, *ad*) = 0.2, *η*(*d*, *cd*) = 0.8, *η*(*c*, *cd*) = 0.9, *η*(*c*, *ac*) = 0.2, *η*(*a*, *ac*) = 0.2} = 0.2; = ∧{*η*(*a*, *ab*) = 0.6, *η*(*b*, *ab*) = 0.5, *η*(*b*, *bc*) = 0.3, *η*(*c*, *bc*) = 0.2, *η*(*c*, *cd*) = 0.9, *η*(*d*, *cd*) = 0.8, *η*(*d*, *ad*) = 0.2, *η*(*a*, *ad*) = 0.2} = 0.2, *respectively. Thus ν*(*a*, *c*) = ∨{0.2, 0.2, 0.2} = 0.2, *this implies that* $\Omega_{a,c}^{\tilde{G}}=0.2$.

**Definition 14**. *Let*$\tilde{G}=\left\{\sigma ,\tau ,\eta \right\}$*be a FIG. Then the* Ω *of* $\tilde{G}$ *is defined as*

$$\Omega \left(\tilde{G}\right)=\vee \left\{\Omega_{l,m}^{\tilde{G}}|l,m\in \sigma^{*}\right\}$$

*That is* Ω *of a FIG* $\tilde{G}$ *is defined as the largest* Ω *of different pairs of vertices of* $\tilde{G}$.

**Example 3**. *Assume*$\tilde{G}=\left\{\sigma ,\tau ,\eta \right\}$*is a FIG given in*Fig 2with σ* = {*a*, *b*, *c*, *d*} *andτ*(*ab*) = 0.7, *τ*(*ac*) = 0.3, *τ*(*ad*) = 0.3, *τ*(*bc*) = 0.3, *τ*(*cd*) = 0.9; *η*(*a*, *ab*) = 0.6, *η*(*b*, *ab*) = 0.5, *η*(*a*, *ac*) = 0.2, *η*(*c*, *ac*) = 0.2, *η*(*a*, *ad*) = 0.2, *η*(*d*, *ad*) = 0.2, *η*(*b*, *bc*) = 0.3, *η*(*c*, *bc*) = 0.2, *η*(*c*, *cd*) = 0.9, *η*(*d*, *cd*) = 0.8. *Then*$\Omega_{a,b}^{\tilde{G}}=0.2,\Omega_{a,c}^{\tilde{G}}=0.2,\Omega_{a,d}^{\tilde{G}}=0.2,\Omega_{b,c}^{\tilde{G}}=0.2,\Omega_{b,d}^{\tilde{G}}=0.2$*and*$\Omega_{c,d}^{\tilde{G}}=0.2$. *Hence*$\Omega \left(\tilde{G}\right)=\vee \{\Omega_{a,b}^{\tilde{G}}=0.2,\Omega_{a,c}^{\tilde{G}}=0.2,\Omega_{a,d}^{\tilde{G}}=0.2,\Omega_{b,c}^{\tilde{G}}=0.2,\Omega_{b,d}^{\tilde{G}}=0.2,\Omega_{c,d}^{\tilde{G}}=0.2\}=0.2$.

Next, we will propose a fascinating result related to FIC in the form of a proposition. We can easily calculate the Ω of any FIC by just applying this result. This proposition will help us to save time and energy. Also, this will be helpful to avoid very long calculations.

**Proposition 3**. *The* Ω *of a FIC* $\tilde{G}$ *is the I*_*s*_ of $\tilde{G}$.

*Proof*. It follows by Proposition 1 that each *I*_*p*_ is a $I_{p}^{s}$ in a FIC. Therefore, the Ω of a FIC $\tilde{G}$ is the *I*_*s*_ of $\tilde{G}$.

Now, we are going to introduce an actual result in the form of a theorem. With the help of this theorem, we will be able to compute Ω of any *CFIG*. By applying this theorem, we do not have to need to do complicated calculations. We have to use the theorem and get the required result.

**Theorem 2**. *Let*$\tilde{G}$*be a CFIG with vertices h*_1_, *h*_2_, …, *h*_*n*_ *such that σ*(*h*_*i*_) = *j*_*i*_ *and j*_1_ ≤ *j*_2_ ≤ …*j*_*n*−2_ ≤ *j*_*n*−1_ ≤ *j*_*n*_ *with τ*(*xy*) = *σ*(*x*) ∧ *σ*(*y*) *for each x*, *y* ∈ σ*. *Then* $\Omega \left(\tilde{G}\right)=j_{n-2}$.

*Proof*. Suppose the conditions of the Theorem. Since any three vertices of $\tilde{G}$ are adjacent because $\tilde{G}$ is a *CFIG* also any three vertices are in 3 vertices FIC. Since $\tilde{G}$ is a *CFIG*, by Proposition 2 all *I*_*p*_ are $I_{p}^{s}$ in *CFIG*. Therefore, to calculate the smallest *I*_*s*_ of FIC in $\tilde{G}$, it is enough to calculate the smallest *I*_*s*_ of every 3 vertices FIC in $\tilde{G}$. Since $\tilde{G}$ is a *CFIG* therefore to examine a 4 vertices FIC, *C* = *abcda* in $\tilde{G}$ (it is notable that the case is same for n vertices FIC) there will be parts of two 3 vertices FIC in *C*, namely *C*_1_ = *abca* and *C*_2_ = *acda*. Let the *I*_*s*_(*C*) = *j*. For all *I*_*p*_ (*x*, *xy*) in *C*, *η*(*x*, *xy*) ≥ *j*. Particularly, *η*(*a*, *ab*) ≥ *j* and *η*(*b*, *bc*) ≥ *j*. Since $\tilde{G}$ is a *CFIG*, then by Proposition 2, $\tilde{G}$ does not have any *δ* − *IPr*. This means *η*(*a*, *ac*) ≥ ∧{*η*(*a*, *ab*), *η*(*b*, *bc*)} ≥ *j*. Thus *η*(*a*, *ac*) ≥ *j*.

Consider, *η*(*a*, *ac*) = *j*, then *I*_*s*_(*C*_1_) = *I*_*s*_(*C*_2_) = *I*_*s*_(*C*) = *j*. Suppose *η*(*a*, *ac*) > *j*, since *I*_*s*_(*C*) = *j* then either *C*_1_ or *C*_2_ will have *I*_*s*_ equal to *j*. Now, *I*_*s*_(*C*) = ∧{*I*_*s*_(*C*_1_), *I*_*s*_(*C*_2_)}. Thus the *I*_*s*_ of a 4 vertices FIC is same as the *I*_*s*_ of a 3 vertices FIC in $\tilde{G}$. From all 3 vertices FIC, the 3 vertices FIC devised by three vertices with largest vertices strength will have the greatest strength. Therefore, the FIC *C* = *h*_*n*−2_, (*h*_*n*−2_, *h*_*n*−2_ *h*_*n*−1_), *h*_*n*−2_ *h*_*n*−1_, (*h*_*n*−1_, *h*_*n*−2_ *h*_*n*−1_), *h*_*n*−1_, (*h*_*n*−1_, *h*_*n*−1_ *h*_*n*_), *h*_*n*−1_ *h*_*n*_, (*h*_*n*_, *h*_*n*−1_ *h*_*n*_), *h*_*n*_, (*h*_*n*_, *h*_*n*_ *h*_*n*−2_), *h*_*n*_ *h*_*n*−2_, (*h*_*n*−2_, *h*_*n*_ *h*_*n*−2_), *h*_*n*−2_ is a FIC with largest *I*_*s*_ in $\tilde{G}$. Also *I*_*s*_ of *C* = *j*_*n*−2_ ∧ *j*_*n*−1_ ∧ *j*_*n*_ = *j*_*n*−2_. Hence $\Omega \left(\tilde{G}\right)=j_{n-2}$.

**Fig 3:**
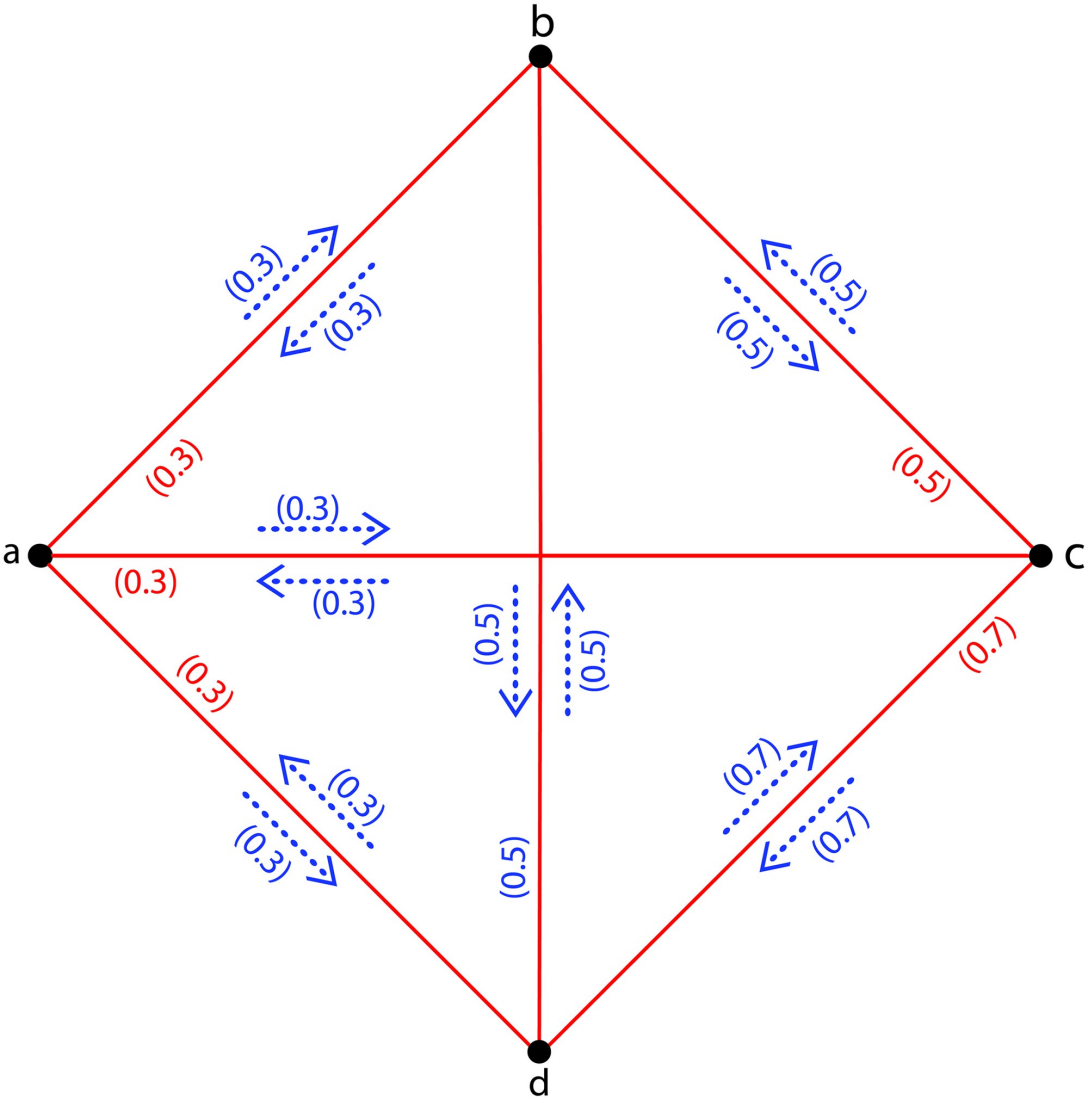
A *CFIG*.

**Example 4**. *Assume*$\tilde{G}=\left\{\sigma ,\tau ,\eta \right\}$*is a CFIG as shown in*Fig 3with σ* = {*a*, *b*, *c*, *d*} *and τ*(*ab*) = 0.3, *τ*(*ac*) = 0.3, *τ*(*ad*) = 0.3, *τ*(*bc*) = 0.5, *τ*(*bd*) = 0.5, *τ*(*cd*) = 0.7; *η*(*a*, *ab*) = 0.3, *η*(*b*, *ab*) = 0.3, *η*(*a*, *ac*) = 0.3, *η*(*c*, *ac*) = 0.3, *η*(*a*, *ad*) = 0.3, *η*(*d*, *ad*) = 0.3, *η*(*b*, *bc*) = 0.5, *η*(*c*, *bc*) = 0.5, *η*(*b*, *bd*) = 0.5, *η*(*d*, *bd*) = 0.5, *η*(*c*, *cd*) = 0.7, *η*(*d*, *cd*) = 0.7. *Then*$\Omega_{a,b}^{\tilde{G}}=0.3,\Omega_{a,c}^{\tilde{G}}=0.3,\Omega_{a,d}^{\tilde{G}}=0.3,\Omega_{b,c}^{\tilde{G}}=0.5,\Omega_{b,d}^{\tilde{G}}=0.5$*and*$\Omega_{c,d}^{\tilde{G}}=0.5$. *Hence*$\Omega \left(\tilde{G}\right)=\vee \left\{\Omega_{a,b}^{\tilde{G}}=0.3,\Omega_{a,c}^{\tilde{G}}=0.3,\Omega_{a,d}^{\tilde{G}}=0.3,\Omega_{b,c}^{\tilde{G}}=0.5,\Omega_{b,d}^{\tilde{G}}=0.5,\Omega_{c,d}^{\tilde{G}}=0.5\right\}=0.5$. *Theorem 2 is verified*.

**Definition 15**. *A vertex l in a FIG*$\tilde{G}$*s said to be a FICCV if*

$$\Omega \left(\tilde{G}-l\right)<\Omega \left(\tilde{G}\right).$$

**Definition 16**. *An edge* (*l*, *m*) *in a FIG* $\tilde{G}$ *is said to be a FICB if*

$$\Omega \left(\tilde{G}-\left(l,m\right)\right)<\Omega \left(\tilde{G}\right).$$

**Definition 17**. *A pair* (*l*, *lm*) *in a FIG* $\tilde{G}$ *is said to be a FICCP if*

$$\Omega \left(\tilde{G}-\left(l,lm\right)\right)<\Omega \left(\tilde{G}\right).$$

**Definition 18**. *A FIG*$\tilde{G}$*is called cyclically balanced if*$\tilde{G}$*is without FICCV, FICB and FICCP*.

**Fig 4:**
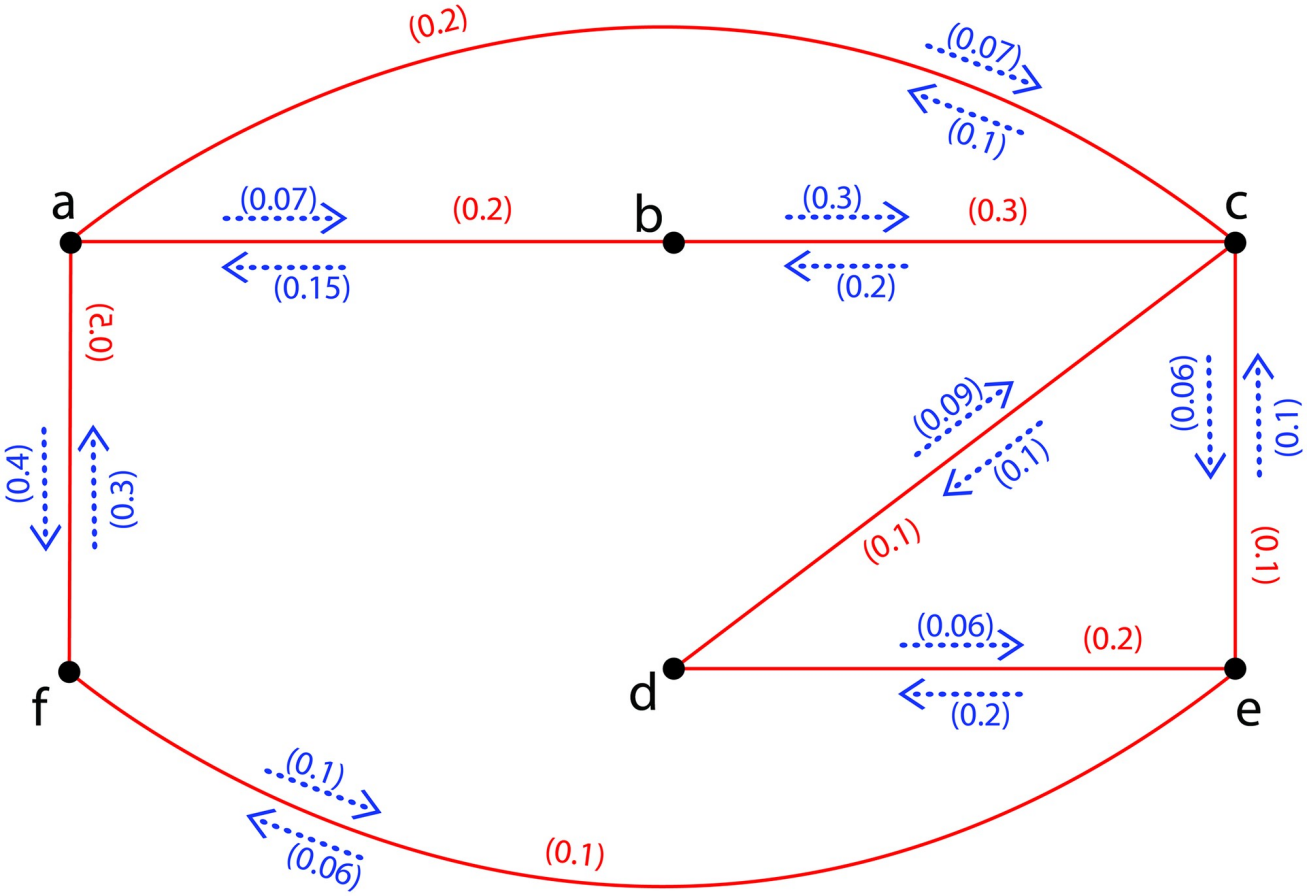
A FIG with *FICCVs*, *FICBs* and *FICCPs*.

**Example 5**. *Assume*$\tilde{G}=\left\{\sigma ,\tau ,\eta \right\}$*is a FIG as provided in*Fig 4with σ* = {*a*, *b*, *c*, *d*, *e*, *f*} *and τ*(*ab*) = 0.2, *τ*(*ac*) = 0.2, *τ*(*af*) = 0.5, *τ*(*bc*) = 0.3, *τ*(*cd*) = 0.1, *τ*(*ce*) = 0.1, *τ*(*de*) = 0.2, *τ*(*ef*) = 0.1; *η*(*a*, *ab*) = 0.07, *η*(*b*, *ab*) = 0.15, *η*(*a*, *ac*) = 0.07, *η*(*c*, *ac*) = 0.1, *η*(*a*, *af*) = 0.4, *η*(*f*, *af*) = 0.3, *η*(*c*, *cd*) = 0.1, *η*(*d*, *cd*) = 0.09, *η*(*c*, *ce*) = 0.06, *η*(*e*, *ce*) = 0.1, *η*(*d*, *de*) = 0.06, *η*(*e*, *de*) = 0.2, *η*(*e*, *ef*) = 0.06, *η*(*f*, *ef*) = 0.1. *Now*, $\Omega_{a,b}^{\tilde{G}}=0.07,\Omega_{a,c}^{\tilde{G}}=0.07,\Omega_{a,d}^{\tilde{G}}=0.06,\Omega_{a,e}^{\tilde{G}}=0.06,\Omega_{a,f}^{\tilde{G}}=0.06,\Omega_{b,c}^{\tilde{G}}=0.07,\Omega_{b,d}^{\tilde{G}}=0.06,\Omega_{b,e}^{\tilde{G}}=0.06,\Omega_{b,f}^{\tilde{G}}=0.06,\Omega_{c,d}^{\tilde{G}}=0.06,\Omega_{c,e}^{\tilde{G}}=0.06,\Omega_{c,f}^{\tilde{G}}=0.06,\Omega_{d,e}^{\tilde{G}}=0.06,\Omega_{d,f}^{\tilde{G}}=0.06,$*and*$\Omega_{e,f}^{\tilde{G}}=0.06$. *Hence*$\Omega (\tilde{G})=0.07$. *Since*$\Omega \left(\tilde{G}-a\right)=\Omega \left(\tilde{G}-b\right)=\Omega \left(\tilde{G}-c\right)=0.06<\Omega \left(\tilde{G}\right)=0.07$. *Therefore*, *a*, *b and c are FICCV*. *In the same way*, $\Omega \left(\tilde{G}-\left(a,b\right)\right)=\Omega \left(\tilde{G}-\left(a,c\right)\right)=\Omega \left(\tilde{G}-\left(b,c\right)\right)=0.06<\Omega (\tilde{G})=0.07$*therefore edges* (*a*, *b*), (*a*, *c*) *and* (*b*, *c*) *re FICBs. Similarly*, (*a*, *ab*), (*b*, *ab*), (*a*, *ac*), (*c*, *ac*), (*b*, *bc*) *and* (*c*, *bc*) *are FICCPs*.

**Proposition 4**. *If an edge* (*l*, *m*) *in a FIG is an FICB, then both the vertices l and m are FICCVs*.

*Proof*. Let $\tilde{G}=\left(\sigma ,\tau ,\eta \right)$ be a FIG and (*l*, *m*) be a *FICB* in $\tilde{G}$. Then by definition of *FICB*, $\Omega \left(\tilde{G}-\left(l,m\right)\right)<\Omega \left(\tilde{G}\right)$. Hence $\Omega \left(\tilde{G}-l\right)\leq \Omega \left(\tilde{G}-\left(l,m\right)\right)<\Omega \left(\tilde{G}\right)$ and $\Omega \left(\tilde{G}-m\right)\leq \Omega \left(\tilde{G}-\left(l,m\right)\right)<\Omega \left(\tilde{G}\right)$. This implies that both vertices *l* and *m* are *FICCVs*.

Now, we are going to establish a handy result for *CFIG*. With the help of this result, we can easily find the *FICCV* in any *CFIG*.

**Theorem 3**. *Let*$\tilde{G}$*be a CFIG with* |σ*| ≥ 4. *Let h*_1_, *h*_2_, …, *h*_*n*_ ∈ σ* *and σ*(*h*_*i*_) = *k*_*i*_ *for i* = 1, 2, …, *n*, *and k*_1_ ≤ *k*_2_ ≤ … ≤ *k*_*n*_. *Then* $\tilde{G}$ *has a FICCV if and only if k*_*n*−3_ < *k*_*n*−2_.

*Proof*. Let *h*_1_, *h*_2_, …, *h*_*n*_ ∈ σ* and *σ*(*h*_*i*_) = *k*_*i*_ for *i* = 1, 2, …, *n*, and *k*_1_ ≤ *k*_2_ ≤ … ≤ *k*_*n*_. Assume that $\tilde{G}$ has a *FICCV* say *l*. Then by definition of *FICCV*, $\Omega \left(\tilde{G}-l\right)<\Omega \left(\tilde{G}\right)$. That is *l* belongs to a distinctive FIC *C*. Suppose *I*_*s*_ of FIC, *C* = λ > *I*_*s*_ of *C*′ for any other FIC, *C*′ in $\tilde{G}$. Since *k*_1_ ≤ *k*_2_ ≤ … ≤ *k*_*n*_, it means that the *I*_*s*_ of the FIC *h*_*n*−2_ *h*_*n*−1_ *h*_*n*−3_ is λ. Hence *l* ∈ {*h*_*n*−2_, *h*_*n*−1_, *h*_*n*−3_}.

To show that *k*_*n*−3_ < *k*_*n*−2_. Assume that *k*_*n*−3_ = *k*_*n*−2_. Then *C*_1_ = *h*_*n*_ *h*_*n*−1_ *h*_*n*−2_ and *C*_2_ = *h*_*n*_ *h*_*n*−1_ *h*_*n*−3_ have the equal *I*_*s*_, and hence the deleting of *h*_*n*−2_, *h*_*n*−1_ or *h*_*n*−3_ will not lessen $\Omega (\tilde{G})$. This contradiction shows that *k*_*n*−3_ < *k*_*n*−2_.

Conversely, assume that *k*_*n*−3_ < *k*_*n*−2_. Now, we have to show that $\tilde{G}$ has a *FICCV*. Since *k*_*n*−2_ ≤ *k*_*n*−1_ ≤ … ≤ *k*_*n*_ and *k*_*n*−3_ < *k*_*n*−2_, all FICs of $\tilde{G}$ have *I*_*s*_ less than that of *I*_*s*_ of *h*_*n*−2_ *h*_*n*−1_ *h*_*n*−3_. Hence the removal of *h*_*n*−1_, *h*_*n*−2_ or *h*_*n*−3_ will become the cause of reduction of $\Omega (\tilde{G})$ therefore, $\tilde{G}$ has a *FICCV*.

## 4 Average cyclic connectivity index of fuzzy incidence graph

Now, we are going to initiate a new idea of *CCI* and *ACCI* of a FIG. The *CCI* of any FIG is denoted by Ω_*I*_. In this section, we formulate the formula to compute the Ω_*I*_ of a FIG. It is also shown that Ω_*I*_ of any *PFISG* $\tilde{H}$ is always ≤ to Ω_*I*_ of any strong fuzzy incidence graph (*SFIG*) $\tilde{G}$. A lower and upper bound of Ω_*I*_ of a *CFIG* is also provided.

**Definition 19**. *In a FIG*$\tilde{G}$, *the* Ω_*I*_ *is defined as*

$$\Omega_{I}\left(\tilde{G}\right)=\sum_{l,m\in \sigma^{*}}\sigma \left(l\right)\sigma \left(m\right)\Omega_{l,m}^{\tilde{G}},$$

*where* $\Omega_{l,m}^{\tilde{G}}$ *is the* Ω *between vertices l and m in* $\tilde{G}$.

**Fig 5:**
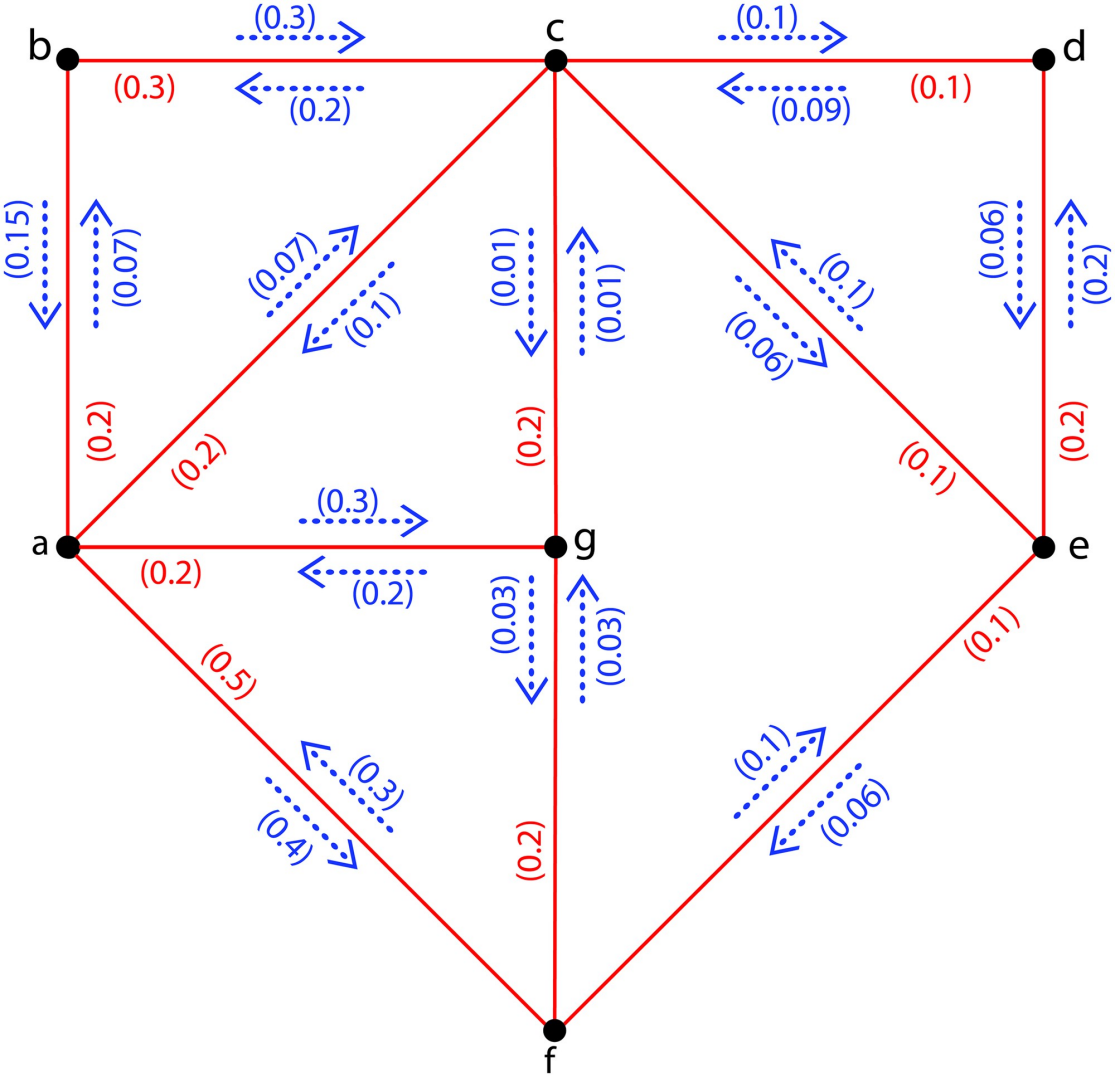
A FIG with Ω_*I*_ = 1.14.

**Example 6**. Fig 5*is indicating the FIG*$\tilde{G}=\left\{\sigma ,\tau ,\eta \right\}$with σ* = {*a*, *b*, *c*, *d*, *e*, *f*, *g*} *and τ*(*ab*) = 0.2, *τ*(*ac*) = 0.2, *τ*(*af*) = 0.5, *τ*(*ag*) = 0.2, *τ*(*bc*) = 0.3, *τ*(*cd*) = 0.1, *τ*(*ce*) = 0.1, *τ*(*cg*) = 0.2, *τ*(*de*) = 0.2, *τ*(*ef*) = 0.1, *τ*(*fg*) = 0.2; *η*(*a*, *ab*) = 0.07, *η*(*b*, *ab*) = 0.15, *η*(*a*, *ac*) = 0.07, *η*(*c*, *ac*) = 0.1, *η*(*a*, *af*) = 0.4, *η*(*f*, *af*) = 0.3, *η*(*a*, *ag*) = 0.2, *η*(*g*, *ag*) = 0.3, *η*(*b*, *bc*) = 0.3, *η*(*c*, *bc*) = 0.2, *η*(*c*, *cd*) = 0.1, *η*(*d*, *cd*) = 0.09, *η*(*c*, *ce*) = 0.06, *η*(*e*, *ce*) = 0.1, *η*(*c*, *cg*) = 0.01, *η*(*g*, *cg*) = 0.01, *η*(*d*, *de*) = 0.06, *η*(*e*, *de*) = 0.2, *η*(*e*, *ef*) = 0.06, *η*(*f*, *ef*) = 0.1, *η*(*f*, *fg*) = 0.03, *η*(*g*, *fg*) = 0.03. *Thus by computing*, $\Omega_{a,b}^{\tilde{G}}=0.07,\Omega_{a,c}^{\tilde{G}}=0.07,\Omega_{a,d}^{\tilde{G}}=0.06,\Omega_{a,e}^{\tilde{G}}=0.06,\Omega_{a,f}^{\tilde{G}}=0.06,\Omega_{a,g}^{\tilde{G}}=0.03,\Omega_{b,c}^{\tilde{G}}=0.07,\Omega_{b,d}^{\tilde{G}}=0.06,\Omega_{b,e}^{\tilde{G}}=0.06,\Omega_{b,f}^{\tilde{G}}=0.06,\Omega_{b,g}^{\tilde{G}}=0.03,\Omega_{c,d}^{\tilde{G}}=0.06,\Omega_{c,e}^{\tilde{G}}=0.06,\Omega_{c,f}^{\tilde{G}}=0.06,\Omega_{c,g}^{\tilde{G}}=0.03,\Omega_{d,e}^{\tilde{G}}=0.06,\Omega_{d,f}^{\tilde{G}}=0.06,\Omega_{d,g}^{\tilde{G}}=0.03,\Omega_{e,f}^{\tilde{G}}=0.06,\Omega_{e,g}^{\tilde{G}}=0.03$*and*$\Omega_{f,g}^{\tilde{G}}=0.06$. *Hence*$\Omega_{I}\left(\tilde{G}\right)=3\left(0.07\right)+13\left(0.06\right)+5\left(0.03\right)=1.14$.

**Remark 4**. *Assume*$\tilde{G}$*is a FIG with vertex set t*. *Then*0≤ΩI(G˜)≤(t2).

**Definition 20**. *A FIG*$\tilde{G}$*is called SFIG if each of its I*_*p*_ is a $I_{p}^{s}$.

**Proposition 5**. *Let*$\tilde{G}=\left(\sigma ,\tau ,\eta \right)$*be a SFIG. Then PFISG*$\tilde{H}=\left(\iota ,\kappa ,\varsigma \right)$*of*$\tilde{G}$, $\Omega_{I}\left(\tilde{H}\right)\leq \Omega_{I}\left(\tilde{G}\right)$.

*Proof*. Assume $\tilde{G}=\left(\sigma ,\tau ,\eta \right)$ is a *SFIG* and let $\tilde{H}=\left(\iota ,\kappa ,\varsigma \right)$ be a *PFISG* of $\tilde{G}=\left(\sigma ,\tau ,\eta \right)$. Let *l*, *m* ∈ ι*. Then by the definition of *PFISG* *ι*(*l*) ≤ *σ*(*l*) and *ν*-estimation of *l* and *m* fulfills the relation $\nu \left(l,m\right){|}_{\tilde{H}}\subseteq \nu \left(l,m\right){|}_{\tilde{G}}$. This implies $\Omega_{l,m}^{\tilde{H}}\leq \Omega_{l,m}^{\tilde{G}}$ and

$$\sum_{l,m\in \iota^{*}}\iota \left(l\right)\iota \left(m\right)\Omega_{l,m}^{\tilde{H}}\leq \sum_{l,m\in \sigma^{*}}\sigma \left(l\right)\sigma \left(m\right)\Omega_{l,m}^{\tilde{G}}$$

Hence,

$$\Omega_{I}\left(\tilde{H}\right)\leq \Omega_{I}\left(\tilde{G}\right).$$

**Fig 6:**
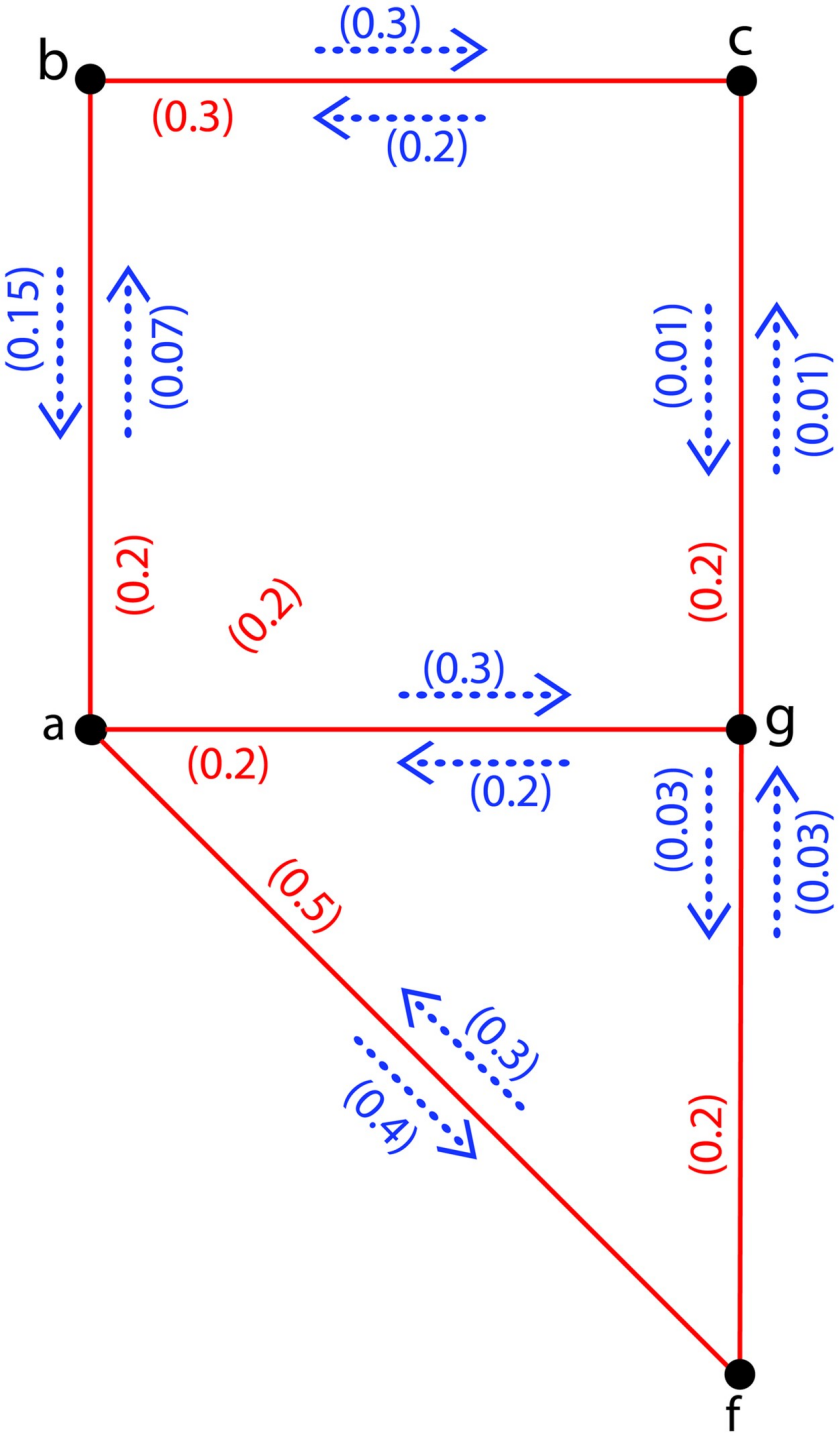
A *PFISG* $\tilde{H}$ with Ω_*I*_ = 0.16.

**Example 7**. *Assume*$\tilde{H}=\left\{\sigma ,\tau ,\eta \right\}$*is a PFISG shown in*Fig 6*of FIG provided in*Fig 5with ι* = {*a*, *b*, *c*, *f*, *g*} *and τ*(*ab*) = 0.2, *τ*(*af*) = 0.5, *τ*(*ag*) = 0.2, *τ*(*bc*) = 0.3, *τ*(*cg*) = 0.2, *τ*(*gf*) = 0.2; *η*(*a*, *ab*) = 0.07, *η*(*b*, *ab*) = 0.15, *η*(*a*, *af*) = 0.4, *η*(*f*, *af*) = 0.3, *η*(*a*, *ag*) = 0.2, *η*(*g*, *ag*) = 0.3, *η*(*b*, *bc*) = 0.3, *η*(*c*, *bc*) = 0.2, *η*(*c*, *cg*) = 0.01, *η*(*g*, *cg*) = 0.01, *η*(*g*, *gf*) = 0.03, *η*(*f*, *gf*) = 0.03. *Now*, $\Omega_{a,b}^{\tilde{H}}=0.01,\Omega_{a,c}^{\tilde{H}}=0.01,\Omega_{a,f}^{\tilde{H}}=0.03,\Omega_{a,g}^{\tilde{H}}=0.03,\Omega_{b,c}^{\tilde{H}}=0.01,\Omega_{b,f}^{\tilde{H}}=0.01,\Omega_{b,g}^{\tilde{H}}=0.01,\Omega_{c,f}^{\tilde{H}}=0.01,\Omega_{c,g}^{\tilde{H}}=0.01$*and*$\Omega_{g,f}^{\tilde{H}}=0.03$. *Hence*$\Omega_{I}\left(\tilde{H}\right)=7\left(0.01\right)+3\left(0.03\right)=0.16<\Omega_{I}\left(\tilde{G}\right)=1.14$.

**Proposition 6**. *In any FIG*$\tilde{G}$, $\Omega_{I}\left(\tilde{G}-l\right)<\Omega_{I}\left(\tilde{G}\right)$, *where l is a FICCV of*$\tilde{G}$.

*Proof*. Consider that *l* is a *FICCV* of $\tilde{G}$. Consequently deletion of *l* will lessen $\Omega (\tilde{G})$. Also $\Omega_{I}\left(\tilde{G}\right)=\sum_{m\in \sigma^{*}\backslash \left\{l\right\}}\sigma \left(l\right)\sigma \left(m\right)\Omega_{l,m}^{\tilde{G}}+\sum_{x,y\in \sigma^{*}\backslash \left\{l\right\}}\sigma \left(x\right)\sigma \left(y\right)\Omega_{x,y}^{\tilde{G}}\geq \sum_{m\in \sigma^{*}\backslash \left\{l\right\}}\sigma \left(l\right)\sigma \left(m\right)\Omega_{l,m}^{\tilde{G}}+\Omega_{I}\left(\tilde{G}-l\right)>\Omega_{I}\left(\tilde{G}-l\right)$. If $\sum_{m\in \sigma^{*}\backslash \left\{l\right\}}\sigma \left(l\right)\sigma \left(m\right)\Omega_{l,m}^{\tilde{G}}>0$, then the statement $\Omega_{I}\left(\tilde{G}\right)=\sum_{m\in \sigma^{*}\backslash \left\{l\right\}}\sigma \left(l\right)\sigma \left(m\right)\Omega_{l,m}^{\tilde{G}}+\sum_{x,y\in \sigma^{*}\backslash \left\{l\right\}}\sigma \left(x\right)\sigma \left(y\right)\Omega_{x,y}^{\tilde{G}}\geq \sum_{m\in \sigma^{*}\backslash \left\{l\right\}}\sigma \left(l\right)\sigma \left(m\right)\Omega_{l,m}^{\tilde{G}}+\Omega_{I}\left(\tilde{G}-l\right)>\Omega_{I}\left(\tilde{G}-l\right)$ is true otherwise it is not true. On the contrary, if $\sum_{m\in \sigma^{*}\backslash \left\{l\right\}}\sigma \left(l\right)\sigma \left(m\right)\Omega_{l,m}^{\tilde{G}}=0$, then $\Omega_{l,m}^{\tilde{G}}=0$ for each *m* ∈ σ*∖{*l*}. This means *l* is not a part of any FIC in $\tilde{G}$ but this is impossible because *l* is a *FICCV* of $\tilde{G}$. Hence we may come to an end that $\sum_{m\in \sigma^{*}\backslash \left\{l\right\}}\sigma \left(l\right)\sigma \left(m\right)\Omega_{l,m}^{\tilde{G}}>0$ now this implies $\Omega_{I}\left(\tilde{G}-l\right)<\Omega_{I}\left(\tilde{G}\right)$.

Now, we are going to discuss lower and upper bounds of Ω_*I*_ of a *CFIG*. For this, we have presented two fundamental theorems. With the help of these theorems, we can quickly know about the Ω_*I*_ of a *CFIG*.

**Theorem 5**. *Assume*$\tilde{G}$*is a CFIG having* |σ*| = *n* ≥ 3, *σ*(*h*_*i*_) = *k*_*i*_ *for i* = 1, 2, …, *n and 0* < *σ*(*h*_*i*_) < 1. *Then* $\Omega_{I}\left(\tilde{G}\right)<\vee \left\{\sigma \left(h_{i}\right)\right\}$.

*Proof*. Since $\tilde{G}$ is a *CFIG* with |σ*| = *n* ≥ 3 therefore by Proposition 2 $\tilde{G}$ does not have any *δ* − *IPr*. This means all *I*_*p*_ in $\tilde{G}$ are $I_{p}^{s}$ and according to Proposition 1 each FIC is a strong FIC in $\tilde{G}$. Suppose *l* and *m* are any two vertices of $\tilde{G}$ then we have to calculate *I*_*s*_ of all FICs contain vertices *l* and *m*. After this, we have to compute Ω which is the maximum value of *I*_*s*_ of all FICs containing pair of vertices *l* and *m*. Similarly, we have to compute Ω up to *n* (total number of vertices) and take the minimum value of all Ω of the *CFIG* $\tilde{G}$. It is clear that $\Omega_{I}\left(\tilde{G}\right)$ can never be exceed the maximum membership value of all the vertices by definition of $\Omega_{I}\left(\tilde{G}\right)=\sum_{l,m\in \sigma^{*}}\sigma \left(l\right)\sigma \left(m\right)\Omega_{l,m}^{\tilde{G}}$. Also, multiplication of all Ω to the membership values of the corresponding vertices and addition of all these Ω of the graph $\tilde{G}$ provide the $\Omega_{I}\left(\tilde{G}\right)$ less than ∨{*σ*(*h*_*i*_)}. This implies that $\Omega_{I}\left(\tilde{G}\right)<\vee \left\{\sigma \left(h_{i}\right)\right\}$.

**Fig 7:**
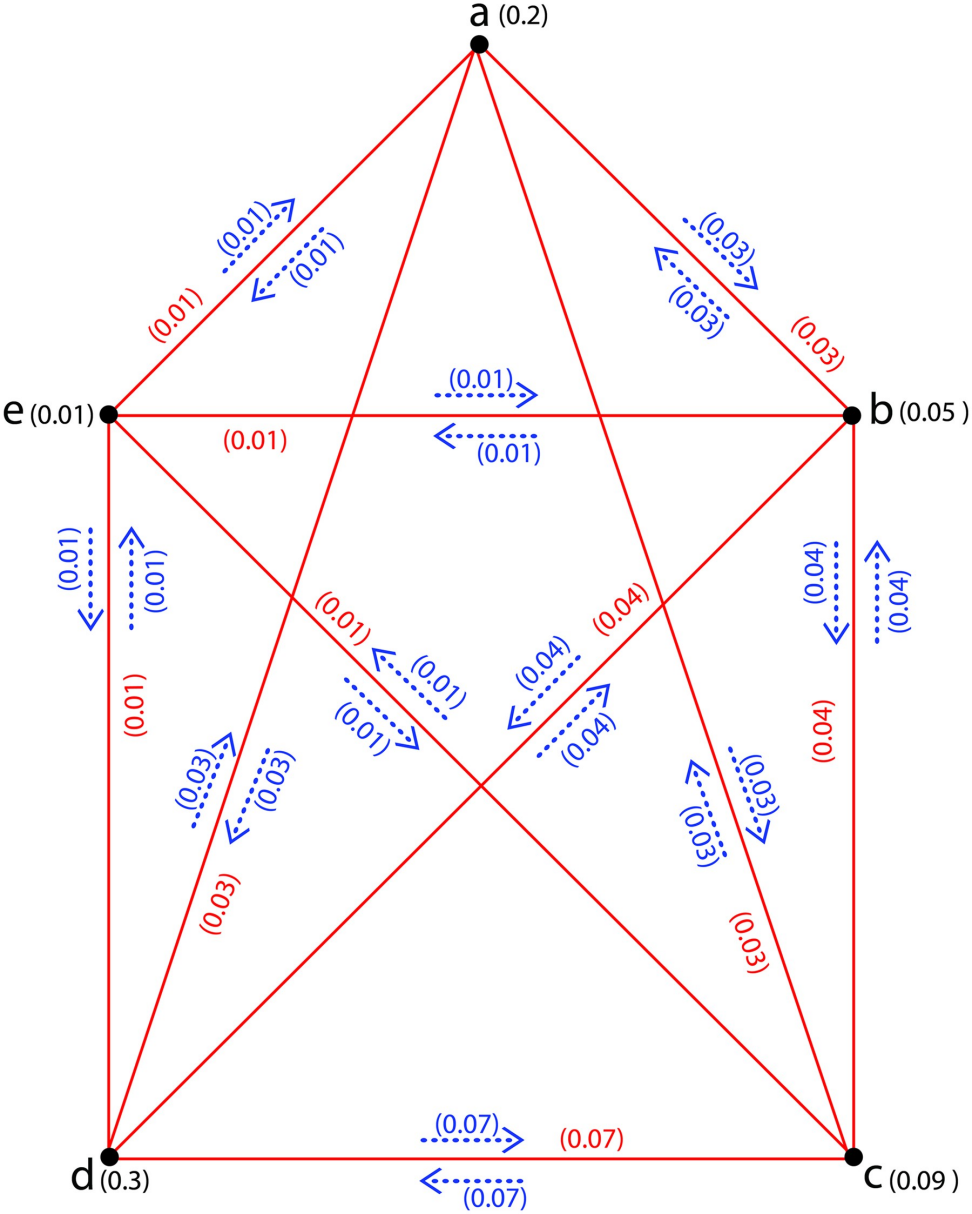
A *CFIG* with (n2)(∧(Ω))3=.00001≤ΩI(G˜)=.004564≤(n2)(∨(σi))3=0.27.

**Example 8**. *Assume*$\tilde{G}=\left\{\sigma ,\tau ,\eta \right\}$*is a CFIG as given in*Fig 7with σ* = {*a*, *b*, *c*, *d*, *e*} *and σ*(*a*) = 0.2, *σ*(*b*) = 0.05, *σ*(*c*) = 0.09, *σ*(*d*) = 0.3, *σ*(*e*) = 0.01; *τ*(*ab*) = 0.03, *τ*(*ac*) = 0.03, *τ*(*ad*) = 0.03, *τ*(*ae*) = 0.01, *τ*(*bc*) = 0.04, *τ*(*bd*) = 0.04, *τ*(*be*) = 0.01, *τ*(*cd*) = 0.07, *τ*(*ce*) = 0.01, *τ*(*de*) = 0.01; *η*(*a*, *ab*) = 0.03, *η*(*b*, *ab*) = 0.03, *η*(*a*, *ac*) = 0.03, *η*(*c*, *ac*) = 0.03, *η*(*a*, *ad*) = 0.03, *η*(*d*, *ad*) = 0.03, *η*(*a*, *ae*) = 0.01, *η*(*e*, *ae*) = 0.01, *η*(*b*, *bc*) = 0.04, *η*(*c*, *bc*) = 0.04, *η*(*b*, *bd*) = 0.04, *η*(*d*, *bd*) = 0.04, *η*(*b*, *be*) = 0.01, *η*(*e*, *be*) = 0.01*η*(*c*, *cd*) = 0.07, *η*(*d*, *cd*) = 0.07, *η*(*c*, *ce*) = 0.01, *η*(*e*, *ce*) = 0.01, *η*(*d*, *de*) = 0.01, *η*(*e*, *de*)0.01. *Then*$\Omega_{I}\left(\tilde{G}\right)=.004564<\vee \left\{0.2,0.05,0.09,0.3,0.01\right\}=0.3$.

**Theorem 6**. *Let*$\tilde{G}$*be a CFIG having* |σ*| = *n* ≥ 3, *σ*(*h*_*i*_) = *k*_*i*_ *for i* = 1, 2, …, *n*. *Then*

(n2)(∧(Ω))3≤ΩI(G˜)≤(n2)(∨(σi))3.

*Proof*. By given statement of theorem. $\tilde{G}$ is a *CFIG* with |σ*| = *n* ≥ 3 therefore Proposition 2 yields that $\tilde{G}$ will be without any *δ* − *IPr*. This means all *I*_*p*_ in $\tilde{G}$ are $I_{p}^{s}$ and as stated in Proposition 1 every FIC is a strong FIC in $\tilde{G}$. Suppose *l* and *m* are any two vertices of $\tilde{G}$ then we have to calculate *I*_*s*_ of all FICs contain vertices *l* and *m*. After this, we have to compute Ω which is the maximum value of *I*_*s*_ of all FICs containing pair of vertices *l* and *m*. Similarly, we have to compute Ω up to *n* (total number of vertices) and take the minimum value of all Ω of the *CFIG* $\tilde{G}$. Also, the total number of edges for a *CFIG* is always equal to (n2)=n!(n-2)!2!. Since $\Omega_{I}\left(\tilde{G}\right)=\sum_{l,m\in \sigma^{*}}\sigma \left(l\right)\sigma \left(m\right)\Omega_{l,m}^{\tilde{G}}$ therefore, (n2)(∧(Ω))3 can never be exceed than the $\Omega_{I}\left(\tilde{G}\right)$. This implies,

(n2)(∧(Ω))3≤ΩI(G˜) (1)

Now, by definition of $\Omega_{I}\left(\tilde{G}\right)=\sum_{l,m\in \sigma^{*}}\sigma \left(l\right)\sigma \left(m\right)\Omega_{l,m}^{\tilde{G}}$ it can be seen that $\Omega_{I}\left(\tilde{G}\right)$ will always remains less than or equal to the (n2)(∨(σi))3. This means

ΩI(G˜)≤(n2)(∨(σi))3 (2)

Hence from Eqs (1) and (2) it can be concluded that

(n2)(∧(Ω))3≤ΩI(G˜)≤(n2)(∨(σi))3.

**Example 9**. *A CFIG*$\tilde{G}=\left\{\sigma ,\tau ,\eta \right\}$*is shown in*Fig 7withσ* = {*a*, *b*, *c*, *d*, *e*} *and σ*(*a*) = 0.2, *σ*(*b*) = 0.05, *σ*(*c*) = 0.09, *σ*(*d*) = 0.3, *σ*(*e*) = 0.01; *τ*(*ab*) = 0.03, *τ*(*ac*) = 0.03, *τ*(*ad*) = 0.03, *τ*(*ae*) = 0.01, *τ*(*bc*) = 0.04, *τ*(*bd*) = 0.04, *τ*(*be*) = 0.01, *τ*(*cd*) = 0.07, *τ*(*ce*) = 0.01, *τ*(*de*) = 0.01; *η*(*a*, *ab*) = 0.03, *η*(*b*, *ab*) = 0.03, *η*(*a*, *ac*) = 0.03, *η*(*c*, *ac*) = 0.03, *η*(*a*, *ad*) = 0.03, *η*(*d*, *ad*) = 0.03, *η*(*a*, *ae*) = 0.01, *η*(*e*, *ae*) = 0.01, *η*(*b*, *bc*) = 0.04, *η*(*c*, *bc*) = 0.04, *η*(*b*, *bd*) = 0.04, *η*(*d*, *bd*) = 0.04, *η*(*b*, *be*) = 0.01, *η*(*e*, *be*) = 0.01*η*(*c*, *cd*) = 0.07, *η*(*d*, *cd*) = 0.07, *η*(*c*, *ce*) = 0.01, *η*(*e*, *ce*) = 0.01, *η*(*d*, *de*) = 0.01, *η*(*e*, *de*)0.01. *Then*(n2)(∧(Ω))3=.00001≤ΩI(G˜)=.004564≤(n2)(∨(σi))3=0.27.

Here, we are going to present a very foundational concept of *ACCI* of a FIG. In enormous networks, the sturdy flow among different vertices is mandatory to sustain trustability and devotedness. To guarantee the firmness of the exchange of data in the complete or portion of the network, measuring the average value of the cyclic data exchange is vital. Therefore, we discuss the *ACCI* of a FIG. The *ACCI* of any FIG is denoted by Ω_*AI*_. Also, we define three different types of vertices.

**Definition 21**. *Assume a FIG*$\tilde{G}=\left(\sigma ,\tau ,\eta \right)$. *Then the* Ω_*AI*_ *is defined by*

ΩAI(G˜)=1(t2)∑l,m∈σ*σ(l)σ(m)Ωl,mG˜,

*where t is the number of vertices in* $\tilde{G}$.

**Definition 22**. *Consider a FIG*$\tilde{G}=\left(\sigma ,\tau ,\eta \right)$. *Then a vertex y is known as CCIV if*$\Omega_{AI}\left(\tilde{G}\right)<\Omega_{AI}\left(\tilde{G}-y\right)$. *If*$\Omega_{AI}\left(\tilde{G}\right)=\Omega_{AI}\left(\tilde{G}-y\right)$, *then y is said to be CNV. If*$\Omega_{AI}\left(\tilde{G}\right)>\Omega_{AI}\left(\tilde{G}-y\right)$*then y is named as CCDV*.

In a FIG an isolated vertex is always a *CNV*.

**Fig 8:**
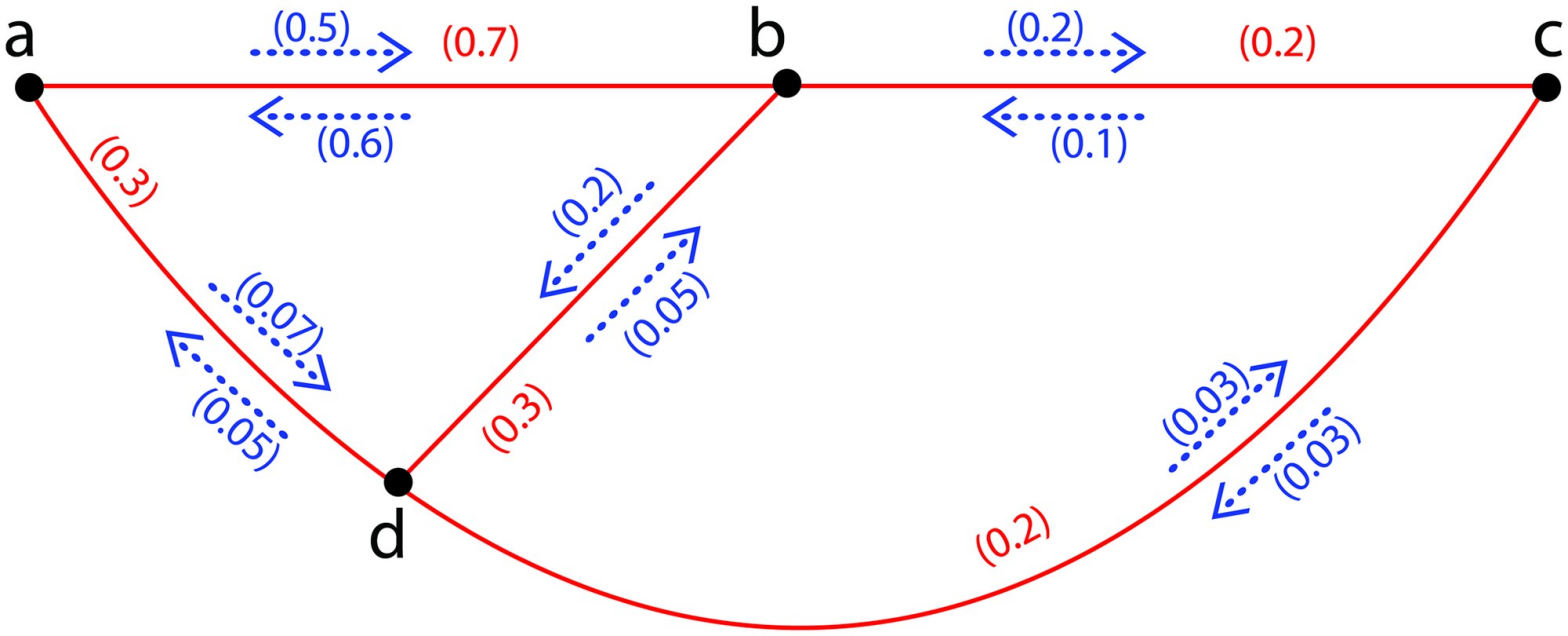
A FIG with *CCDV* and *CCIV*.

**Example 10**. *In*Fig 8*a FIG*$\tilde{G}=\left\{\sigma ,\tau ,\eta \right\}$is provided with σ* = {*a*, *b*, *c*, *d*} *and τ*(*ab*) = 0.7, *τ*(*ad*) = 0.3, *τ*(*bc*) = 0.2, *τ*(*bd*) = 0.3, *τ*(*cd*) = 0.2; *η*(*a*, *ab*) = 0.5, *η*(*b*, *ab*) = 0.6, *η*(*a*, *ad*) = 0.07, *η*(*d*, *ad*) = 0.05, *η*(*b*, *bc*) = 0.2, *η*(*c*, *bc*) = 0.1, *η*(*b*, *bd*) = 0.2, *η*(*d*, *bd*) = 0.05, *η*(*c*, *cd*) = 0.03, *η*(*d*, *cd*) = 0.03. *There are three FICs in*$\tilde{G}$*namely*, *C*_1_: *a*, *b*, *d*, *a*, *C*_2_: *b*, *c*, *d*, *c and C*_3_: *a*, *b*, *c*, *d*, *a with I*_*s*_*are* 0.05, 0.03 *and* 0.03 *respectively. Now*, $\Omega_{a,b}^{\tilde{G}}=0.05,\Omega_{a,c}^{\tilde{G}}=0.03,\Omega_{a,d}^{\tilde{G}}=0.05,\Omega_{b,c}^{\tilde{G}}=0.03,\Omega_{b,d}^{\tilde{G}}=0.05$ *and* $\Omega_{c,d}^{\tilde{G}}=0.03$. *Therefore*, $\Omega_{I}\left(\tilde{G}\right)=0.03+0.03+0.05+0.03+0.05+0.03=0.22$. *Also* ΩAI(G˜)=1(t2)∑l,m∈σ*σ(l)σ(m)Ωl,mG˜=1(42)0.22=0.036. *Also after calculation* $\Omega_{AI}\left(\tilde{G}-a\right)=0.03<\Omega_{AI}\left(\tilde{G}\right)=0.036\Omega_{AI}\left(\tilde{G}-b\right)=0<\Omega_{AI}\left(\tilde{G}\right)=0.036$,$\Omega_{AI}\left(\tilde{G}-d\right)=0<\Omega_{AI}\left(\tilde{G}\right)=0.036$ *and* $\Omega_{AI}\left(\tilde{G}-c\right)=0.05>\Omega_{AI}\left(\tilde{G}\right)=0.036$. *Now*, *a*, *b and d are CCDV and c is a CCIV*.

Now, we are going to present an essential proposition. With the help of this proposition, we will be able to find *CNV*, *CCIV*, and *CCDV* of FIG.

**Proposition 7**. *Let*$\tilde{G}=\left(\sigma ,\tau ,\eta \right)$*be a FIG having* ∣σ*∣ = *t* ≥ 3 *and assume x* ∈ σ*. *Let* $r=\frac{\Omega_{I}\left(\tilde{G}\right)}{\Omega_{I}\left(\tilde{G}-x\right)}$ *and* $s=\frac{t}{t-2}$. *Then x is said to be a CNV if and only if s* = *r*, *x is named as CCIV if and only if r* < *s and x is called CCDV if and only if r* > *s*.

*Proof*. Consider a FIG $\tilde{G}=\left(\sigma ,\tau ,\eta \right)$ having ∣σ*∣ = *t* ≥ 3 and consider *x* ∈ σ*. Suppose that *x* is a *CCIV*, then by definition of *CCIV*, the $\Omega_{AI}\left(\tilde{G}-x\right)>\Omega_{AI}\left(\tilde{G}\right)$. Clearly, ΩI(G˜-x)(t-12)>ΩI(G˜)(t2) and also we have $\frac{\Omega_{I}\left(\tilde{G}-x\right)}{t-2}>\frac{\Omega_{I}\left(\tilde{G}\right)}{t}$. Thus, $\frac{\Omega_{I}\left(\tilde{G}\right)}{\Omega_{I}\left(\tilde{G}-x\right)}<\frac{t}{t-2}$. If $r=\frac{\Omega_{I}\left(\tilde{G}\right)}{\Omega_{I}\left(\tilde{G}-x\right)}$ and $s=\frac{t}{t-2}$, then *r* < *s*. Converse part is trivial. Therefore, *x* is *CCIV if and only if r* < *s*. Remaining cases may be proved in a similar way.

## 5 Real-life applications of cycle connectivity

In daily life, Ω has various uses. Here, we have proposed two critical real-life applications of Ω of FIGs. In the first application, we have taken a highway system of different cities and apply the idea of Ω of FIG to find the roads which are becoming the leading cause of maximum accidents. In the second application, we have taken a network of different computers sharing data. We have applied the idea of Ω to the network of different computers and find which computer/computers are transferring the maximum amount of data to other computers.

### 5.1 Application of cycle connectivity in highway system

Due to the huge traffic on roads, the percentage of accidents is increasing day by day. To minimize these accidents government should take some serious steps to lessen the percentage of road accidents. Here, we are presenting a graphical model of FIG to tackle this problem. This can be done by calculating the Ω between each pair of vertices of FIG. The roads having a maximum Ω are the roads of maximum traffic flow and become a source of the highest road accidents. Government can work on these roads by making speed breakers, speed bumps and deploying more traffic wardens to minimize road accidents. Here, we include an application of Ω of FIG in a highway system of different cities. As an explanatory case, consider a network of FIG consisting of eight vertices expressing different cities *c*_1_, *c*_2_, *c*_3_, *c*_4_, *c*_5_, *c*_6_, *c*_7_ and *c*_8_, edges are indicating the roads joining these cities and *I*_*p*_ are expressing flow of traffic from one city to another city. For example, an *I*_*p*_ (*c*_1_, *c*_1_ *c*_2_) is showing the flow of traffic from city *c*_1_ to city *c*_2_ and an *I*_*p*_ (*c*_2_, *c*_1_ *c*_2_) is proclaiming the flow of traffic from city *c*_2_ to city *c*_1_ through road *c*_1_ *c*_2_. The membership value (MSV) of the edges is showing the traffic flow (bikes, cars, vehicles, heavy vehicles) among different cities and the MSV of (*c*_1_, *c*_1_ *c*_2_) is specifying the flow of traffic from city *c*_1_ to city *c*_2_ and the MSV of (*c*_2_, *c*_1_ *c*_2_) is proclaiming the flow of traffic from city *c*_2_ to city *c*_1_ through road *c*_1_ *c*_2_.

**Fig 9:**
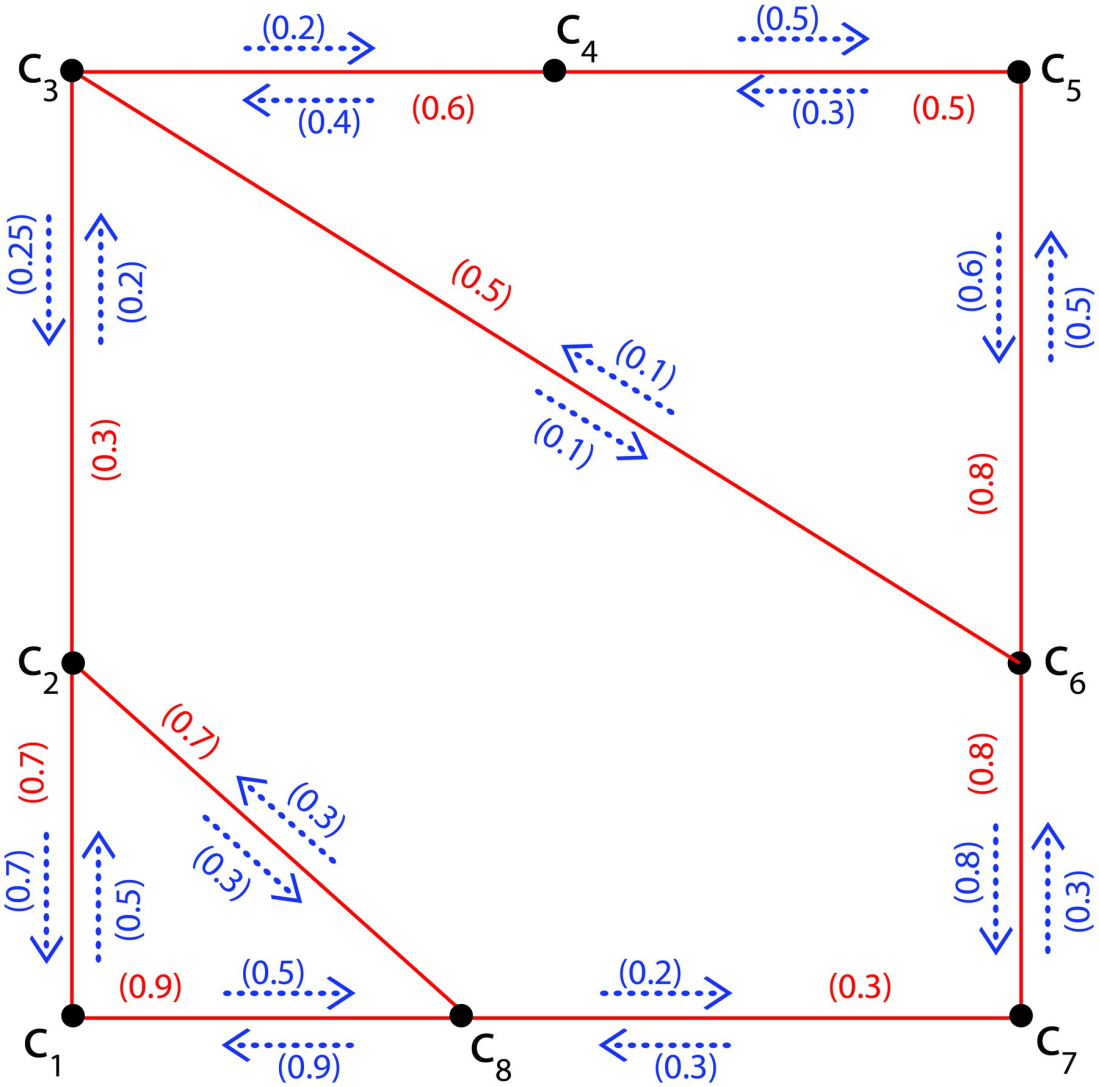
A highway system of different cities.

Assume $\tilde{G}=\left(\sigma ,\tau ,\eta \right)$ is a FIG as shown in Fig 9 representing a highway system with σ* = {*c*_1_, *c*_2_, *c*_3_, *c*_4_, *c*_5_, *c*_6_, *c*_7_, *c*_8_} and *τ*(*c*_1_ *c*_2_) = 0.7, *τ*(*c*_1_ *c*_8_) = 0.9, *τ*(*c*_2_ *c*_3_) = 0.3, *τ*(*c*_2_ *c*_8_) = 0.7, *τ*(*c*_3_ *c*_4_) = 0.6, *τ*(*c*_3_ *c*_6_) = 0.5, *τ*(*c*_4_ *c*_5_) = 0.5, *τ*(*c*_5_ *c*_6_) = 0.8, *τ*(*c*_6_ *c*_7_) = 0.8, *τ*(*c*_7_ *c*_8_) = 0.3; *η*(*c*_1_, *c*_1_ *c*_2_) = 0.5, *η*(*c*_2_, *c*_1_ *c*_2_) = 0.7, *η*(*c*_1_, *c*_1_ *c*_8_) = 0.5, *η*(*c*_8_, *c*_1_ *c*_8_) = 0.9, *η*(*c*_2_, *c*_2_ *c*_3_) = 0.2, *η*(*c*_3_, *c*_2_ *c*_3_) = 0.25, *η*(*c*_2_, *c*_2_ *c*_8_) = 0.3, *η*(*c*_8_, *c*_2_ *c*_8_) = 0.3, *η*(*c*_3_, *c*_3_ *c*_4_) = 0.2, *η*(*c*_4_, *c*_3_ *c*_4_) = 0.4, *η*(*c*_3_, *c*_3_ *c*_6_) = 0.1, *η*(*c*_6_, *c*_3_ *c*_6_) = 0.1, *η*(*c*_4_, *c*_4_ *c*_5_) = 0.5, *η*(*c*_5_, *c*_4_ *c*_5_) = 0.3, *η*(*c*_5_, *c*_5_ *c*_6_) = 0.6, *η*(*c*_6_, *c*_5_ *c*_6_) = 0.5, *η*(*c*_6_, *c*_6_ *c*_7_) = 0.8, *η*(*c*_7_, *c*_6_ *c*_7_) = 0.3, *η*(*c*_7_, *c*_7_ *c*_8_) = 0.3, *η*(*c*_8_, *c*_7_ *c*_8_) = 0.2. There are eight FICs in $\tilde{G}$ namely, *C*_1_: *c*_1_, *c*_2_, *c*_8_, *c*_1_, *C*_2_: *c*_1_, *c*_2_, *c*_3_, *c*_6_, *c*_7_, *c*_8_, *c*_1_, *C*_3_: *c*_1_, *c*_2_, *c*_3_, *c*_4_, *c*_5_, *c*_6_, *c*_7_, *c*_8_, *c*_1_, *C*_4_: *c*_3_, *c*_4_, *c*_5_, *c*_6_, *c*_3_, *C*_5_: *c*_3_, *c*_4_, *c*_5_, *c*_6_, *c*_7_, *c*_8_, *c*_2_, *c*_3_, *C*_6_: *c*_3_, *c*_4_, *c*_5_, *c*_6_, *c*_7_, *c*_8_, *c*_1_, *c*_2_, *c*_3_, *C*_7_: *c*_3_, *c*_6_, *c*_7_, *c*_8_, *c*_2_, *c*_3_, and *C*_8_: *c*_3_, *c*_6_, *c*_7_, *c*_8_, *c*_1_, *c*_2_, *c*_3_. Then $\Omega_{c_{1},c_{2}}^{\tilde{G}}=\Omega_{c_{1},c_{8}}^{\tilde{G}}=\Omega_{c_{2},c_{8}}^{\tilde{G}}=0.3$ and $\Omega_{c_{1},c_{3}}^{\tilde{G}}=\Omega_{c_{1},c_{4}}^{\tilde{G}}=\Omega_{c_{1},c_{5}}^{\tilde{G}}=\Omega_{c_{1},c_{6}}^{\tilde{G}}=\Omega_{c_{1},c_{7}}^{\tilde{G}}=\Omega_{c_{2},c_{3}}^{\tilde{G}}=\Omega_{c_{2},c_{4}}^{\tilde{G}}=\Omega_{c_{2},c_{5}}^{\tilde{G}}=\Omega_{c_{2},c_{6}}^{\tilde{G}}=\Omega_{c_{2},c_{7}}^{\tilde{G}}=\Omega_{c_{3},c_{4}}^{\tilde{G}}=\Omega_{c_{3},c_{5}}^{\tilde{G}}=\Omega_{c_{3},c_{6}}^{\tilde{G}}=\Omega_{c_{3},c_{7}}^{\tilde{G}}=\Omega_{c_{3},c_{8}}^{\tilde{G}}=\Omega_{c_{4},c_{5}}^{\tilde{G}}=\Omega_{c_{4},c_{6}}^{\tilde{G}}=\Omega_{c_{4},c_{7}}^{\tilde{G}}=\Omega_{c_{4},c_{8}}^{\tilde{G}}=\Omega_{c_{5},c_{6}}^{\tilde{G}}=\Omega_{c_{5},c_{7}}^{\tilde{G}}=\Omega_{c_{5},c_{8}}^{\tilde{G}}=\Omega_{c_{6},c_{7}}^{\tilde{G}}=\Omega_{c_{6},c_{8}}^{\tilde{G}}=\Omega_{c_{7},c_{8}}^{\tilde{G}}=0.2$. Hence $\Omega \left(\tilde{G}\right)=\vee \left\{\Omega_{c_{1},c_{2}}^{\tilde{G}}=\Omega_{c_{1},c_{8}}^{\tilde{G}}=\Omega_{c_{2},c_{8}}^{\tilde{G}}=0.3,\Omega_{c_{1},c_{3}}^{\tilde{G}}=\Omega_{c_{1},c_{4}}^{\tilde{G}}=\Omega_{c_{1},c_{5}}^{\tilde{G}}=\Omega_{c_{1},c_{6}}^{\tilde{G}}=\Omega_{c_{1},c_{7}}^{\tilde{G}}=\Omega_{c_{2},c_{3}}^{\tilde{G}}=\Omega_{c_{2},c_{4}}^{\tilde{G}}=\Omega_{c_{2},c_{5}}^{\tilde{G}}=\Omega_{c_{2},c_{6}}^{\tilde{G}}=\Omega_{c_{2},c_{7}}^{\tilde{G}}=\Omega_{c_{3},c_{4}}^{\tilde{G}}=\Omega_{c_{3},c_{5}}^{\tilde{G}}=\Omega_{c_{3},c_{6}}^{\tilde{G}}=\Omega_{c_{3},c_{7}}^{\tilde{G}}=\Omega_{c_{3},c_{8}}^{\tilde{G}}=\Omega_{c_{4},c_{5}}^{\tilde{G}}=\Omega_{c_{4},c_{6}}^{\tilde{G}}=\Omega_{c_{4},c_{7}}^{\tilde{G}}=\Omega_{c_{4},c_{8}}^{\tilde{G}}=\Omega_{c_{5},c_{6}}^{\tilde{G}}=\Omega_{c_{5},c_{7}}^{\tilde{G}}=\Omega_{c_{5},c_{8}}^{\tilde{G}}=\Omega_{c_{6},c_{7}}^{\tilde{G}}=\Omega_{c_{6},c_{8}}^{\tilde{G}}=\Omega_{c_{7},c_{8}}^{\tilde{G}}=0.2\right\}=0.3$. Thus $\Omega (\tilde{G})=0.3$ is representing that the roads joining cities *c*_1_ *c*_2_, *c*_1_ *c*_8_ and *c*_2_ *c*_8_ are the main roads which are becoming a main cause of highest percentage of road accidents. So, the government should focus on these roads by making more speed breakers, speed bumps and deploying more traffic wardens on these roads. In this way, they can minimize the percentage of road accidents.

We have used FIGs in our application. The FIGs are more instrumental and effective than graphs. We cannot use graphs to explain the above phenomenon because graphs do not show the impact of a vertex on an edge. Another thing, in graphs, the Ω between each pair of vertices is always equal to 1, and we are unable to find which roads are becoming the main cause of maximum road accidents, but in FIGs, the Ω between each pair of vertices will be different. Therefore, FIGs are more helpful and useful than graphs.

### 5.2 Application of cycle connectivity in a computer network

In a network of different computers, computers are sharing data with each other. We want to find which computer/computers are best in performance among all other computers and sharing maximum data with all other computers in a network. This can be done by computing Ω between each pair of computers in a network. The pair of computers which have a maximum Ω will be the required computers transferring maximum data to all other computers in a network. Here, we are presenting a graphical model of FIG to explain this phenomenon. As an example, assume a network of FIG comprising of eight vertices. The vertices are showing the eight distinct computers in a network. The MSV of the vertices is indicating data store in each of these computers, the MSV of the edges is demonstrating the total amount of data that can be transferred from one computer to another computer and the MSV of the *I*_*p*_ is representing the amount of data which one computer is transferring to another computer. For example, an *I*_*p*_ (*a*, *ab*) is indicating the transfer of data from computer *a* to computer *b* and an *I*_*p*_ (*b*, *ab*) is showing the transfer of data from computer *b* to computer *a*.

**Fig 10:**
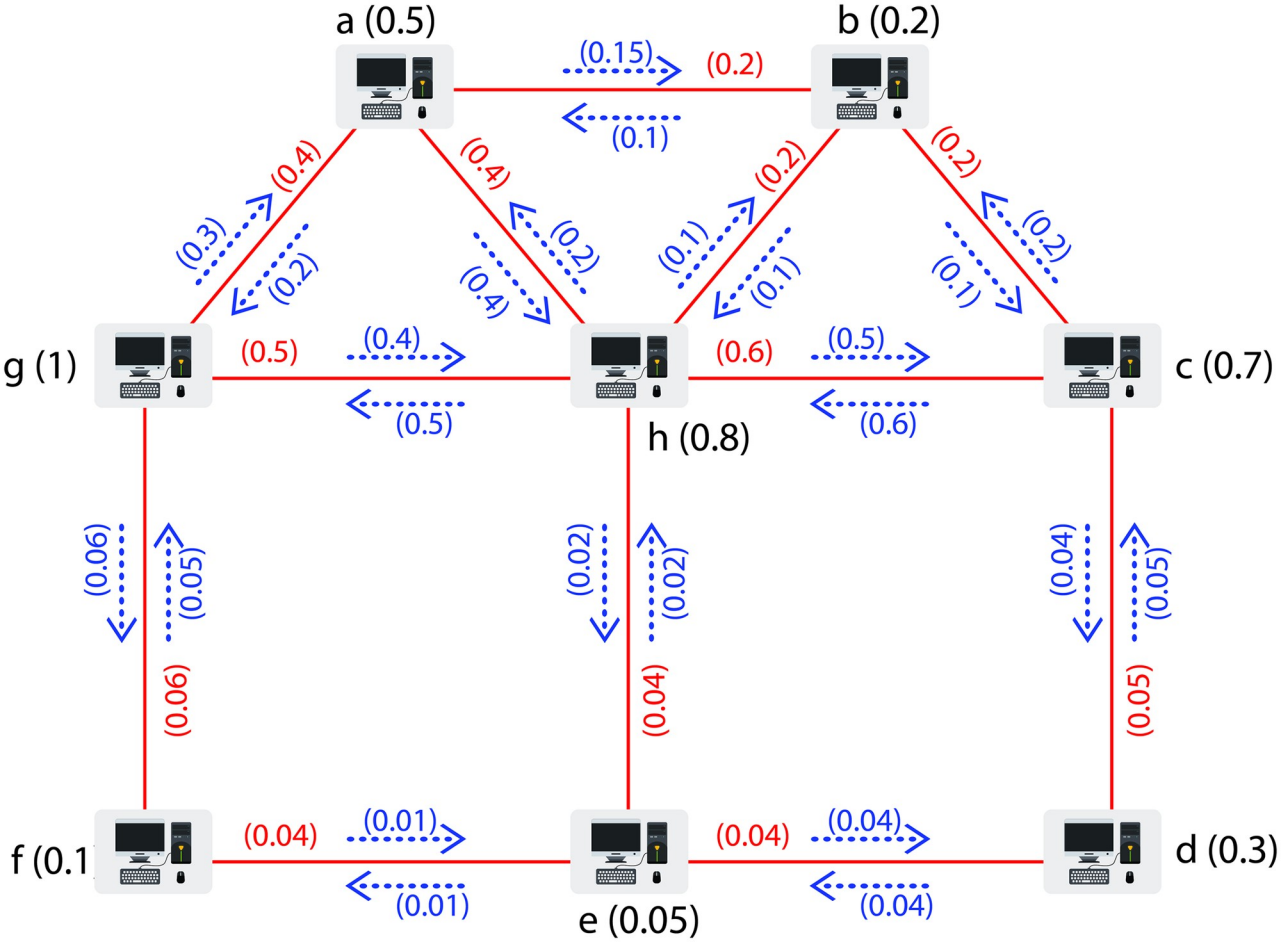
A network of different computers.

Let $\tilde{G}=\left(\sigma ,\tau ,\eta \right)$ be a FIG as shown in Fig 10 representing a network of different computers with *σ*(*a*) = 0.5, *σ*(*b*) = 0.2, *σ*(*c*) = 0.7, *σ*(*d*) = 0.3, *σ*(*e*) = 0.05, *σ*(*f*) = 0.1, *σ*(*g*) = 1, *σ*(*h*) = 0.8; *τ*(*ab*) = 0.2, *τ*(*ag*) = 0.4, *τ*(*ah*) = 0.4, *τ*(*bc*) = 0.2, *τ*(*bh*) = 0.2, *τ*(*cd*) = 0.05, *τ*(*ch*) = 0.6, *τ*(*de*) = 0.04, *τ*(*ef*) = 0.04, *τ*(*eh*) = 0.04, *τ*(*fg*) = 0.06, *τ*(*gh*) = 0.5; *η*(*a*, *ab*) = 0.15, *η*(*b*, *ab*) = 0.1, *η*(*a*, *ag*) = 0.2, *η*(*g*, *ag*) = 0.3, *η*(*a*, *ah*) = 0.4, *η*(*h*, *ah*) = 0.2, *η*(*b*, *bc*) = 0.1, *η*(*c*, *bc*) = 0.2, *η*(*b*, *bh*) = 0.1, *η*(*h*, *bh*) = 0.1, *η*(*c*, *cd*) = 0.04, *η*(*d*, *cd*) = 0.05, *η*(*c*, *ch*) = 0.6, *η*(*h*, *ch*) = 0.5, *η*(*d*, *de*) = 0.04, *η*(*e*, *de*) = 0.04, *η*(*e*, *ef*) = 0.01, *η*(*f*, *ef*) = 0.01, *η*(*e*, *eh*) = 0.02, *η*(*h*, *eh*) = 0.02, *η*(*f*, *fg*) = 0.05, *η*(*g*, *fg*) = 0.06, *η*(*g*, *gh*) = 0.4, *η*(*h*, *gh*) = 0.5. Now, by computing Ω between each pair of vertices we get $\Omega_{a,b}^{\tilde{G}}=\Omega_{a,c}^{\tilde{G}}=\Omega_{a,g}^{\tilde{G}}=\Omega_{a,h}^{\tilde{G}}=\Omega_{b,c}^{\tilde{G}}=\Omega_{b,g}^{\tilde{G}}=\Omega_{b,h}^{\tilde{G}}=\Omega_{c,g}^{\tilde{G}}=\Omega_{c,h}^{\tilde{G}}=0.1,\Omega_{a,d}^{\tilde{G}}=\Omega_{a,e}^{\tilde{G}}=\Omega_{b,d}^{\tilde{G}}=\Omega_{b,e}^{\tilde{G}}=\Omega_{c,d}^{\tilde{G}}=\Omega_{c,e}^{\tilde{G}}=\Omega_{d,e}^{\tilde{G}}=\Omega_{d,g}^{\tilde{G}}=\Omega_{d,h}^{\tilde{G}}=\Omega_{e,g}^{\tilde{G}}=\Omega_{e,h}^{\tilde{G}}=0.02,\Omega_{a,f}^{\tilde{G}}=\Omega_{b,f}^{\tilde{G}}=\Omega_{c,f}^{\tilde{G}}=\Omega_{d,f}^{\tilde{G}}=\Omega_{e,f}^{\tilde{G}}=\Omega_{f,g}^{\tilde{G}}=\Omega_{f,h}^{\tilde{G}}=0.01$ and $\Omega_{g,h}^{\tilde{G}}=0.2$. Thus $\Omega_{g,h}^{\tilde{G}}=0.2$ is representing the maximum Ω between computers *g* and *h*. Therefore, computers *g* and *h* are best computers in performance among all other computers and sharing maximum data with all other computers in a network.

## 6 Comparative analysis

Here, we are going to compare our model with the existing model. In Fig 9 a FIG is indicating a highway system of eight different cities *c*_1_, *c*_2_, *c*_3_, *c*_4_, *c*_5_, *c*_6_, *c*_7_ and *c*_8_ in which the edges are indicating the roads joining these cities and *I*_*p*_ are expressing flow of traffic from one city to another city. For example, an *I*_*p*_ (*c*_1_, *c*_1_ *c*_2_) is showing the flow of traffic from city *c*_1_ to city *c*_2_ and an *I*_*p*_ (*c*_2_, *c*_1_ *c*_2_) is showing the flow of traffic from city *c*_2_ to city *c*_1_ through road *c*_1_ *c*_2_. The MSV of the edges is showing the traffic flow (bikes, cars, vehicles, heavy vehicles) among different cities and the MSV of (*c*_1_, *c*_1_ *c*_2_) is specifying the flow of traffic from city *c*_1_ to city *c*_2_ and (*c*_2_, *c*_1_ *c*_2_) is proclaiming the flow of traffic from city *c*_2_ to city *c*_1_ through road *c*_1_ *c*_2_. Now, if we assign MSVs to all the edges and all *I*_*p*_ of FIG provided in Fig 9 is equal to 1 we get a graph. In the case of graph the Ω between each pair of vertices is $\Omega_{c_{1},c_{2}}^{\tilde{G}}=\Omega_{c_{1},c_{8}}^{\tilde{G}}=\Omega_{c_{2},c_{8}}^{\tilde{G}}=\Omega_{c_{1},c_{3}}^{\tilde{G}}=\Omega_{c_{1},c_{4}}^{\tilde{G}}=\Omega_{c_{1},c_{5}}^{\tilde{G}}=\Omega_{c_{1},c_{6}}^{\tilde{G}}=\Omega_{c_{1},c_{7}}^{\tilde{G}}=\Omega_{c_{2},c_{3}}^{\tilde{G}}=\Omega_{c_{2},c_{4}}^{\tilde{G}}=\Omega_{c_{2},c_{5}}^{\tilde{G}}=\Omega_{c_{2},c_{6}}^{\tilde{G}}=\Omega_{c_{2},c_{7}}^{\tilde{G}}=\Omega_{c_{3},c_{4}}^{\tilde{G}}=\Omega_{c_{3},c_{5}}^{\tilde{G}}=\Omega_{c_{3},c_{6}}^{\tilde{G}}=\Omega_{c_{3},c_{7}}^{\tilde{G}}=\Omega_{c_{3},c_{8}}^{\tilde{G}}=\Omega_{c_{4},c_{5}}^{\tilde{G}}=\Omega_{c_{4},c_{6}}^{\tilde{G}}=\Omega_{c_{4},c_{7}}^{\tilde{G}}=\Omega_{c_{4},c_{8}}^{\tilde{G}}=\Omega_{c_{5},c_{6}}^{\tilde{G}}=\Omega_{c_{5},c_{7}}^{\tilde{G}}=\Omega_{c_{5},c_{8}}^{\tilde{G}}=\Omega_{c_{6},c_{7}}^{\tilde{G}}=\Omega_{c_{6},c_{8}}^{\tilde{G}}=\Omega_{c_{7},c_{8}}^{\tilde{G}}=1$. Since in case of graph the Ω between each pair of vertices is equal to 1. Therefore, we are unable to find the roads which are becoming a main reason of maximum accidents. Hence our model is better than the previous one.

Similarly, in Fig 10 a FIG is representing a network of different computers. Computers are sharing data. We want to find which computer/computers are best in performance among all other computers and sharing maximum data with all other computers in a network. This can be done by computing Ω between each pair of computers in a network. The pair of computers which have a maximum Ω will be the required computers transferring maximum data to all other computers in a network. The vertices are showing the eight distinct computers in a network. The MSV of the vertices is indicating data store in each of these computers, the MSV of the edges is demonstrating the total amount of data that can be transferred from one computer to another computer and the MSV of the *I*_*p*_ is representing the amount of data which one computer is transferring to another computer. For example, an *I*_*p*_ (*a*, *ab*) is indicating the transfer of data from computer *a* to computer *b* and an *I*_*p*_ (*b*, *ab*) is showing the transfer of data from computer *b* to computer *a*.

Now, if we assign MSVs to all the edges and all *I*_*p*_ of the FIG shown in Fig 10 we get a graph. In the case of graph the Ω between each pair of vertices is $\Omega_{a,b}^{\tilde{G}}=\Omega_{a,c}^{\tilde{G}}=\Omega_{a,g}^{\tilde{G}}=\Omega_{a,h}^{\tilde{G}}=\Omega_{b,c}^{\tilde{G}}=\Omega_{b,g}^{\tilde{G}}=\Omega_{b,h}^{\tilde{G}}=\Omega_{c,g}^{\tilde{G}}=\Omega_{c,h}^{\tilde{G}}=\Omega_{a,d}^{\tilde{G}}=\Omega_{a,e}^{\tilde{G}}=\Omega_{b,d}^{\tilde{G}}=\Omega_{b,e}^{\tilde{G}}=\Omega_{c,d}^{\tilde{G}}=\Omega_{c,e}^{\tilde{G}}=\Omega_{d,e}^{\tilde{G}}=\Omega_{d,g}^{\tilde{G}}=\Omega_{d,h}^{\tilde{G}}=\Omega_{e,g}^{\tilde{G}}=\Omega_{e,h}^{\tilde{G}}=\Omega_{a,f}^{\tilde{G}}=\Omega_{b,f}^{\tilde{G}}=\Omega_{c,f}^{\tilde{G}}=\Omega_{d,f}^{\tilde{G}}=\Omega_{e,f}^{\tilde{G}}=\Omega_{f,g}^{\tilde{G}}=\Omega_{f,h}^{\tilde{G}}=$ and $\Omega_{g,h}^{\tilde{G}}=1$. Therefore, pervious model is not helpful to find which computer/computers are transferring maximum amount of data. Thus, our model is more effective and beneficial than the previous one.

## 7 Conclusion

In this article, we advanced the theory of FIGs. The notion of connectivity is indivisible from the theory of FIGs. There are a variety of parameters that command the connectivity of a network. In this article, the authors attempted to make up a new connectivity idea named as Ω, *FICCV*, *FICB*, and *FICCP* in FIGs. Ω of various FIG theoretic structures are examined. As the number of FICs grows, the cyclic accessibility from one vertex to another vertex enhances. This benefits to upgrading the trustability of any network. The formula to compute Ω_*I*_ of any FIG is discussed with a variety of examples. The Ω_*I*_ and Ω_*AI*_ of FIGs are two frameworks associated with the cyclic accessibility of networks. Ω_*AI*_ represents the average strength of the cyclic flow in a network. *CCIV*, *CNV*, and *CCDV* three different types of vertices are also introduced. The criterion to check *CCIV*, *CNV*, and *CCDV* is developed. An application of Ω of FIG in highway systems of different cities to reduce road accidents and in a computer network to find the best computers among all other computers in a network is also provided. A comparative analysis of our study with the existing study is also provided. More related ideas will be contemplated in the upcoming papers.
